# EDMD-Causing Emerin Mutant Myogenic Progenitors Exhibit Impaired Differentiation Using Similar Mechanisms

**DOI:** 10.3390/cells9061463

**Published:** 2020-06-15

**Authors:** Ashvin Iyer, James M. Holaska

**Affiliations:** 1 Department of Biomedical Sciences, Cooper Medical School of Rowan University, Camden, NJ 08103, USA; aiyer@mail.usciences.edu; 2 Department of Pharmaceutical Sciences, University of the Sciences in Philadelphia, Philadelphia, PA 19104, USA

**Keywords:** emerin, Emery–Dreifuss muscular dystrophy, myogenic differentiation, histone deacetylase, nuclear envelope

## Abstract

Mutations in the gene encoding emerin (*EMD*) cause Emery–Dreifuss muscular dystrophy (EDMD1), an inherited disorder characterized by progressive skeletal muscle wasting, irregular heart rhythms and contractures of major tendons. The skeletal muscle defects seen in EDMD are caused by failure of muscle stem cells to differentiate and regenerate the damaged muscle. However, the underlying mechanisms remain poorly understood. Most EDMD1 patients harbor nonsense mutations and have no detectable emerin protein. There are three EDMD-causing emerin mutants (S54F, Q133H, and Δ95–99) that localize correctly to the nuclear envelope and are expressed at wildtype levels. We hypothesized these emerin mutants would share in the disruption of key molecular pathways involved in myogenic differentiation. We generated myogenic progenitors expressing wildtype emerin and each EDMD1-causing emerin mutation (S54F, Q133H, Δ95–99) in an emerin-null (EMD^−/y^) background. S54F, Q133H, and Δ95–99 failed to rescue EMD^−/y^ myogenic differentiation, while wildtype emerin efficiently rescued differentiation. RNA sequencing was done to identify pathways and networks important for emerin regulation of myogenic differentiation. This analysis significantly reduced the number of pathways implicated in EDMD1 muscle pathogenesis.

## 1. Introduction

X-linked Emery–Dreifuss muscular dystrophy (EDMD1) is an inherited disorder caused by mutations in EMD, which encodes emerin. EDMD1 is characterized by progressive skeletal muscle wasting, irregular heart rhythms, and contractures of major tendons [1,2,3,4]. Inefficient skeletal muscle regeneration and impaired differentiation of skeletal muscle stem cells contribute to the skeletal muscle wasting observed in EDMD. Most EDMD1 patients have nonsense mutations [5], resulting from base substitutions, small deletions, or insertions [6,7,8,9,10,11]. However, a few missense mutations and in-frame deletions in EMD produce detectable emerin protein [7,10,11,12].

Emerin residues 46–221 is intrinsically disordered [13,14] and contains binding domains for many of its interactors, including lamins, actin, transcription regulators, and HDAC3 [15,16,17,18,19,20,21,22]. There are four disease-causing EMD mutations within this region that result in normal emerin protein expression and localization; these are S54F, Δ95–99, Q133H, and P183H [10,23,24,25,26]. The structural consequences of these mutated forms are not yet known. It is possible these mutant proteins may either modify the 3D structure and conformational plasticity of the protein or disrupt specific emerin modification events, or both. These changes are predicted to disrupt assembly of protein complexes containing emerin, either by affecting partner recognition or by hindering emerin oligomerization [26].

Emerin knockout mice display delayed skeletal muscle regeneration and repair, mild atrioventricular alterations, and motor coordination defects [27,28]. Biopsies of skeletal muscles from EDMD1, EDMD2, and EMD^−/y^ mice revealed compensatory upregulation of skeletal muscle regeneration genes [27,29]. In particular, the transcriptional pathways regulated by retinoblastoma protein (*Rb*) and *MyoD* genes were altered. Rb protein was found to be inappropriately hyperphosphorylated at key developmental stages, including when myoblasts exit the cell cycle to commit towards differentiation [27,29]. Emerin-downregulated C2C12 myoblasts and EMD^−/y^ myogenic progenitors display impaired myogenic differentiation and fail to fuse into myotubes [30,31,32], due to altered temporal activation of myogenic regulatory genes [33] and perturbation of major canonical pathways [34,35]. The coordinated temporal expression of key muscle differentiation genes, including *MyoD*, *Myf5*, *Pax3*, and *Pax7*, is disrupted in EMD^−/y^ myogenic progenitors [36] due to the failure of the genome to reorganize normally during differentiation [27,29,35].

Emerin interacts directly with the core components of the nuclear corepressor (NCoR) complex, which is involved in repressing genes by stably binding chromatin [37]. HDAC3 is part of the NCoR complex and deacetylates specific lysine residues in histone H4 tails. Emerin binds directly to HDAC3 and stimulates its deacetylase activity [15]. EDMD-causing emerin mutants (S54F, Δ95–99, Q133H, P183H) fail to bind HDAC3 [15]. Activation of HDAC3 activity using theophylline, successfully rescued the temporal localization and expression of *Pax3, Pax7, MyoD*, and *Myf5* genes, and rescued myotube formation in EMD^−/y^ myogenic progenitors [30,36]. Inhibition of HDAC3 activity using RGFP966 significantly impaired myosin heavy chain (MyHC) expression and myotube formation in both wildtype and EMD^−/y^ myogenic progenitors [30]. Recent studies using histone acetyltransferase (HAT) inhibitors showed partial rescue of differentiation commitment and successful rescue of myotube formation in EMD^−/y^ myogenic progenitors [38]. These studies support the hypothesis that emerin interacts with HDAC3 to coordinate the transcriptional reprogramming to reorganize the genome required to transcribe genes needed for differentiation commitments and repress genes involved in proliferation.

We previously showed that EMD^−/y^ myogenic progenitors exhibited impaired differentiation [30,34,35]. EMD^−/y^ progenitors failed to exit the cell cycle appropriately, resulting in delayed myoblast commitment and inhibition of myoblast formation. RNA sequencing (RNAseq) showed that EMD^−/y^ myogenic progenitors failed to completely transcriptionally reprogram upon differentiation induction, which signals the progenitors to exit the cell cycle and commit to myotube formation. More than 1600 genes were differentially expressed in EMD^−/y^ myogenic progenitors at this important differentiation transition [34]. Although this study supported a failure in transcriptional reprogramming, it failed to identify the mechanisms responsible for impaired differentiation of EMD^−/y^ progenitors. Studying differentiation in myogenic progenitors containing EDMD1-causing emerin mutants was predicted to narrow down the potential genes and pathways responsible for EDMD pathogenesis. Here we show, for the first time, that EDMD1-causing emerin mutant myogenic progenitors exhibit impaired differentiation. Transcriptional profiling of these EDMD1-causing myogenic progenitors during differentiation significantly narrowed the pathways implicated in the muscle regeneration pathology of EDMD1.

## 2. Materials and Methods

### 2.1. Cell Culture

Myogenic progenitors from H2K Wildtype and EMD^−/y^ mice were obtained from Tatiana Cohen and Terence Partridge (Children’s National Medical Center, Washington, DC, WA, USA) [35]. Proliferating H2Ks were grown and differentiated as previously described [36]. Proliferating myogenic progenitors were grown in proliferative media consisting of 2% chick embryo extract (Accurate Chemical, Westbury, NY, USA), high-glucose DMEM (ThermoFisher Scientific, Waltham, MA, USA) supplemented with 20% heat-inactivated FBS (ThermoFisher Scientific, Waltham, MA, USA), 1% penicillin–streptomycin (ThermoFisher Scientific, Waltham, MA, USA), 2% l-glutamine (ThermoFisher Scientific, Waltham, MA, USA) and 20 units/mL γ-interferon (MilliporeSigma, Burlington, MA, USA). Proliferating cells were plated on gelatin at a density of approximately 650 cells/cm^2^ and grown at 33 °C and 10% CO_2_. Differentiating cells were plated on gelatin at a density of 25,000 cells/cm^2^ in proliferative conditions for 24 h, then switched to differentiation media consisting of DMEM supplemented with 5% horse serum (ThermoFisher Scientific, Waltham, MA, USA) and 2% l-glutamine, and grown at 37 °C and 5% CO_2_. Cells between passage six and twelve were used for all analyses.

### 2.2. Lentiviral Transduction

H2K myogenic progenitors expressing wildtype emerin (+EMD) and EDMD causing emerin mutations (S54F, Q133H, and Δ95–99), an emerin mutation that does not cause the disease (M179), and a vector only control were generated using the following protocol. EMD^−/y^ mouse myogenic progenitors (EMD^−/y^) were seeded at a density of 1000 cells/well in 96-well plates coated with 0.01% gelatin. Cells were incubated at 33 °C and 10% CO_2_ overnight in proliferation media and replaced with infection medium containing lentiviral particles (Genecopoeia, Rockville, MD, USA, #LPP-CS-G0746-Lv105,) at a multiplicity of infection of 350 and 8 µg/mL polybrene (Cyagen Biosciences, Santa Clara, CA, USA). Polybrene is a cationic polymer known to increase lentiviral transduction efficiency [39] by neutralizing the surface charge between the cell surface and the viral particles [40,41]. The infection medium was replaced with fresh growth media after 16–24 h. Cells were allowed to grow for 72 h post-transduction, then transferred to 12-well dishes containing growth media and puromycin (MilliporeSigma, Burlington, MA, USA, #P8833). EMD^−/y^ cells transduced with control vector, S54F and Δ95–99 were selected using 15 µg/mL puromycin. EMD^−/y^ cells transduced with Q133H and M179 vectors were selected using 10 µg/mL puromycin. EMD^−/y^ cells transduced with wildtype emerin (+EMD) was selected using 6 µg/mL. Once the cells were 40–60% confluent, cells were subsequently moved to six-well dishes, followed by transfer to 10 cm dishes, and finally expanded to multiple 15 cm dishes. Cells were kept sparse to ensure cells don’t undergo spontaneous differentiation or senescence. Media containing puromycin was replaced every 2–3 days.

### 2.3. Myogenic Differentiation Immunofluorescence Assay

Wildtype and EMD^–/y^ cells were seeded at a density of 7800 cells per well into 96-well plates or 92,800 cells per well into 12-well plates coated with gelatin. For experiments in 12-well plates, +EMD, Q133H and Δ95–99 cells were plated at a density of 118,800 cells per well coated with gelatin. For 96-well plate experiments, +EMD, vector control, S54F and M179 were plated at a density of 10,000 cells per well coated with gelatin. Cells were incubated overnight in proliferation media. Differentiation was induced by replacing proliferation media with differentiation media (DMEM with high glucose, 5% horse serum, 1% penicillin/streptomycin) and incubating cells at 37 °C and 5% CO_2_.

EdU (5-ethynyl-2′-deoxyuridine, ThermoFisher Scientific, Waltham, MA, USA) was used as a proliferation marker. EdU is a thymidine analog which gets incorporated into newly synthesized DNA during S phase of the cell cycle. Ten micromolar EdU was incubated with cells for 2 h prior to fixing. Cells were fixed at 0, 24, 48, and 72 h post differentiation induction. After fixing, cells were washed three times and were processed according to the manufacturer’s instructions (Click-IT EdU imaging Kit 647, ThermoFisher Scientific, Waltham, MA, USA). Cells were treated with 0.5% Triton-X100 in phosphate-buffered saline (PBS) for 20 min, washed twice with 3% bovine serum albumin in PBS, and treated with a Click-IT EdU reaction cocktail for 30 min. Cells were washed with PBS and then blocked for an hour with 3% BSA in PBS containing 0.1% Triton-X100.

Cells were then treated with emerin antibodies (Santa Cruz Biotechnology, Dallas, TX, USA, #FL-254,) for an hour, washed three times with PBS, and incubated with monoclonal antibodies against MyHC (Santa Cruz Biotechnology, Dallas, TX, USA, #MYH B-5,) for an hour. Cells were washed three times with PBS, incubated with secondary antibodies Alexa Fluor 488 goat anti-rabbit (1:200, ThermoFisher Scientific, Waltham, MA, USA) for one hour followed by Alexa Fluor 594 goat anti-mouse antibodies (1:200, ThermoFisher Scientific, Waltham, MA, USA) for one hour. Cells were washed twice with PBS and incubated with 0.2 µg/mL DAPI to label nuclei. Cells were washed once with PBS and imaged using the EVOS-FL Auto microscope (ThermoFisher Scientific, Waltham, MA, USA). 

Multiple images were acquired at random (at least three) for each well using a 40× objective. A total of three wells were analyzed for each treatment for a given biological replicate. At least two biological replicates were done for each treatment. Each field was imaged in four different channels: green fluorescent protein channel for emerin positive nuclei, blue fluorescent channel for DAPI stained nuclei, Cy5 channel for Edu positive nuclei, and Texas Red channel for MyHC positive nuclei. Image analysis was done using the cell counter plugin in ImageJ. Cell cycle withdrawal was determined by dividing the total number of cells expressing EdU by the total number of nuclei in an image. Differentiation index (DI) was defined as the percentage of nuclei expressing the terminal differentiation MyHC. Myotube formation was determined by measuring the percentage of myotubes containing at least three nuclei and expressing MyHC.

### 2.4. Western Blots

Proliferating H2K myogenic progenitors in 15 cm dishes were harvested using SDS-PAGE sample buffer. A total of 50,000 cell equivalents for all cell lines except for +EMD and M179 expressing cells (100,000 cell equivalents) were separated by SDS-PAGE and transferred onto a nitrocellulose membrane. Blocking of the membrane was done using 5% dried nonfat milk in PBS containing 0.1% Tween 20 (PBST) at room temperature for an hour. The membrane was then incubated with emerin antibodies (rabbit polyclonal, 1:2500, Proteintech, Rosemont, IL, USA) for an hour followed by three washes in PBST for five minutes. The membrane was then incubated for an hour at room temperature with goat anti-rabbit HRP antibodies (1:10,000, ThermoFisher Scientific), followed by three PBST washes for five minutes each. The blot was incubated with Amersham ECL western blotting detection reagents (GE healthcare, Chicago, IL, USA, #RPN2209) and imaged on the Bio-Rad Chemidoc equipment (Bio-Rad Laboratories, Hercules, CA, USA). The HRP enzyme was then inactivated with 0.1% sodium azide in PBST for 15 min followed by five washes (five minutes each) with PBS. The blot was incubated with mouse gamma-tubulin antibodies (1:10,000, ThermoFisher Scientific, Waltham, MA, USA) for an hour at room temperature followed by incubation with donkey anti-mouse HRP antibodies (1:10,000, ThermoFisher Scientific, Waltham, MA, USA) for an hour at room temperature. The blots were then incubated with Amersham ECL western blotting detection reagents (GE healthcare, Chicago, IL, USA, #RPN2209) and processed according to the manufacturer’s instructions. ImageLab software (Bio-Rad Laboratories) was used to perform volume analysis.

### 2.5. RNAseq

For isolation of total RNA during proliferation, each H2K myogenic progenitor cell line was seeded at a density of 158,000 cells per 15 cm dish coated with 0.01% gelatin and incubated in proliferative media for two days. Two 15 cm dishes were combined and represent one technical replicate. Three technical replicates per sample were performed for each biological replicate (*n* = 2). Cells were trypsizined, washed three times with PBS, and total RNA was isolated using the mRNeasy Plus Kit (Qiagen, Germantown, MD, USA, #74136) per manufacturer’s instructions.

Wildtype and EMD^−/y^ H2K myogenic progenitors were seeded in six-well dishes coated with 0.01% gelatin at a density of 232,000 cells/well for isolation of total RNA during differentiation. 297,000 cells/well of vector control and emerin mutant cell lines were plated in six-well dishes coated with 0.01% gelatin. Cells were incubated overnight in proliferative media. In the morning, cell lines were switched to differentiation media. RNA was isolated after 0 h (to account for changes due to cell density), 24 h, 48 h, and 72 h of differentiation. RNA was isolated using the mRNeasy Plus Kit (Qiagen, Germantown, MD, USA, #74136) per manufacturer’s instructions. Three technical replicates per sample were performed for each biological replicate (*n* = 2).

Purity of RNA was assessed using Nanodrop (ND-2000), and quantification was done using the Quant-IT RNA assay kit (ThermoFisher Scientific, Waltham, MA, USA, #Q10213). BGI Genomics performed library preparation and sequencing using BGISEQ-500 platform. BGI Genomics filtered reads using SOAPnuke and genome mapping was done to the mouse genome (mm10) using hierarchical indexing for spliced alignment of Transcripts-2 (HISAT2). Differentially expressed genes was detected using DEseq2. Transcripts were considered to be significantly differentially expressed if the *q*-value <0.05. The *q*-value is a *p*-value corrected for the False Discovery Rate (FDR) and represents the standard in the field for determining significance. All differentially expressed transcripts were also ≥2-fold increased or decreased. KEGG pathway enrichment analysis was done using phyper, a function of R.

### 2.6. Data Sharing Statement

RNA sequencing data is available through the NCBI Gene Expression Omnibus (Accession number GSE152226).

## 3. Results

### 3.1. Generation of Stable Myogenic Progenitor Cell Lines Expressing EDMD-Causing Emerin Mutants

**Figure 1:**
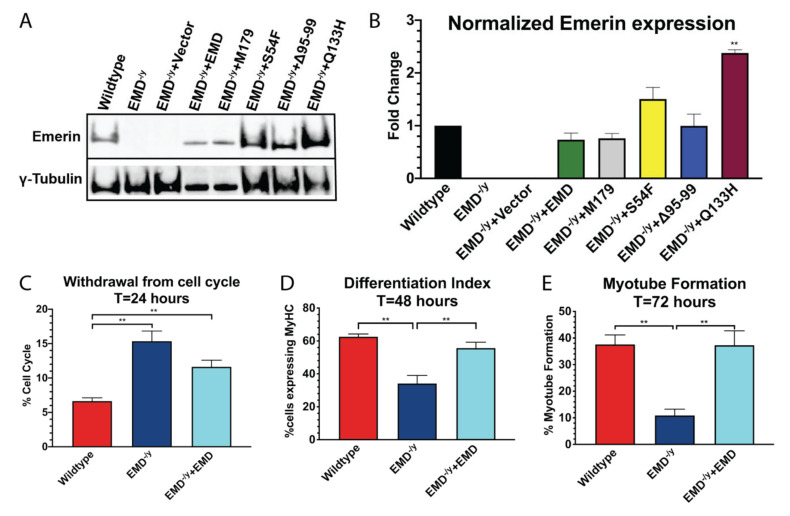
Transduction with emerin rescues differentiation in EMD^−/y^ myogenic progenitors. (**A**) Antibodies against emerin and γ-tubulin confirmed successful expression of lentiviral targets; (**B**) Densitometry was performed, and emerin protein levels in each cell line were normalized to γ-tubulin. Levels of emerin in each cell line was then normalized to wildtype cell line. (**C**) Wildtype (red), EMD^−/y^ (dark blue) and +EMD (cyan) myogenic progenitors were induced to differentiate by serum withdrawal, and withdrawal from cell cycle was monitored at 24 h by measuring the incorporation of EdU; (**D**) The differentiation index was assessed at 48 h by determining the percentage of cells expressing MyHC; (**E**) Myotube formation was determined at 72 h by measuring the percentage of myotubes containing at least 3 nuclei and expressing MyHC; *p < 0.05, **p < 0.02.

Western blotting with antibodies against emerin confirmed successful expression of each of the emerin proteins; γ-tubulin was used as loading control (Figure 1A,B). EMD^−/y^ myogenic progenitor lines expressed wildtype emerin (+EMD; 0.74-fold), Δ95–99 (0.99-fold), S54F (1.51-fold; not significant), and M179 (0.76-fold) at levels similar to wildtype myogenic progenitors. EMD^−/y^ myogenic progenitor lines expressing Q133H had 2.38-fold more protein. EMD^−/y^ and vector alone failed to express any emerin, as expected (Figure 1B).

Differentiation of wildtype, EMD^−/y^, and +EMD myogenic progenitors were assessed to test if wildtype emerin rescued differentiation of EMD^−/y^ progenitors. After 24 h, more than 90% of wildtype progenitors withdrew from the cell cycle, whereas 15.4% of EMD^−/y^ myogenic progenitors were still active in the cell cycle (*p* < 0.02, Figure 1C). +EMD myogenic progenitors displayed a trend towards rescue. In total, 62.6% of differentiating wildtype cells expressed MyHC at 48 h, whereas, 34.1% of EMD^−/y^ cells expressed MyHC (*p* < 0.02; Figure 1D). Expression of wildtype emerin rescued MyHC expression, as +EMD cells showed a differentiation index of 55.7% (Figure 1D). Only 10.9% of EMD^−/y^ myogenic progenitors formed myotubes after 72 h, compared to 37.6% of wildtype progenitors (*p* < 0.02; Figure 1E). Expression of wildtype emerin rescued myotube formation, as 37.3% of +EMD cells successfully formed myotubes (Figure 1E).

### 3.2. Myogenic Progenitors Expressing S54F and M179 Exhibit Impaired Differentiation

**Figure 2:**
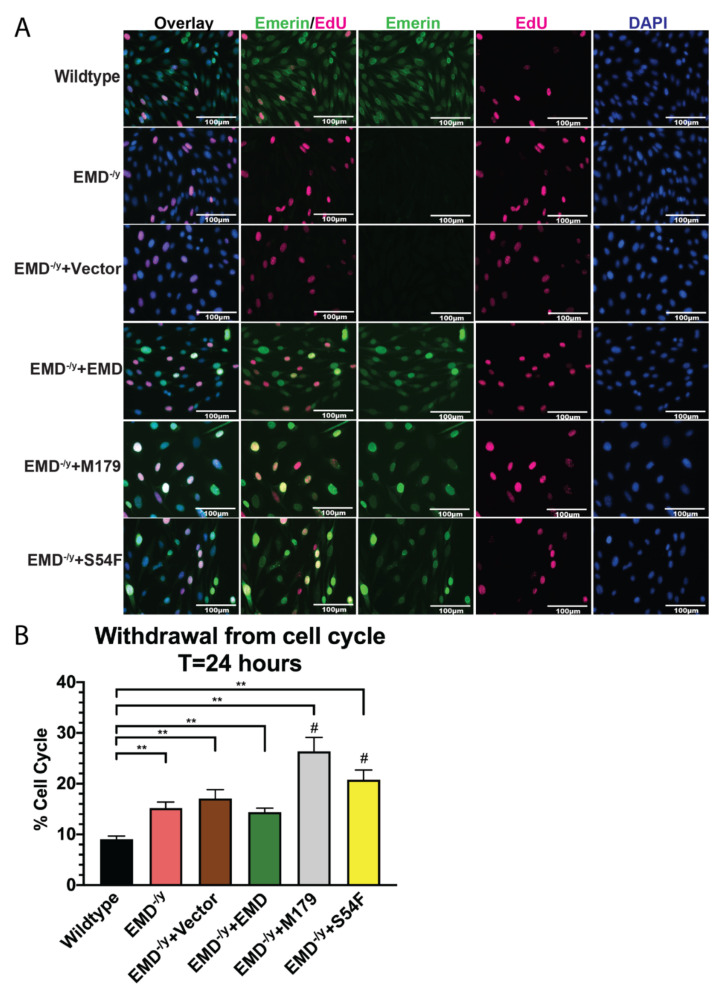
S54F and M179 emerin mutant myogenic progenitors fail to exit the cell cycle. (**A**) Immunofluorescence images at 24 h post differentiation induction. Emerin is shown in green, EdU is shown in pink, and nuclei are blue. Scale bar: 100 µm. (**B**) Cell cycle withdrawal at 24 h post differentiation induction. **p < 0.02; #*p* < 0.05 in comparison to +EMD.

Wildtype, EMD^−/y^, vector alone (control), +EMD, M179, and S54F myogenic progenitor lines were induced to differentiate by serum withdrawal. Cell cycle withdrawal was analyzed after 24 h. 9.1% of wildtype myogenic progenitors incorporated EdU (Figure 2A,B), whereas 15.2% of EMD^−/y^ and 17.1% of control cells incorporated EdU (*p* < 0.02). In total, 14.4% of +EMD cells incorporated EdU. In total, 26.4% of M179 and 20.8% of S54F progenitors incorporated EdU (Figure 2A,B).

**Figure 3:**
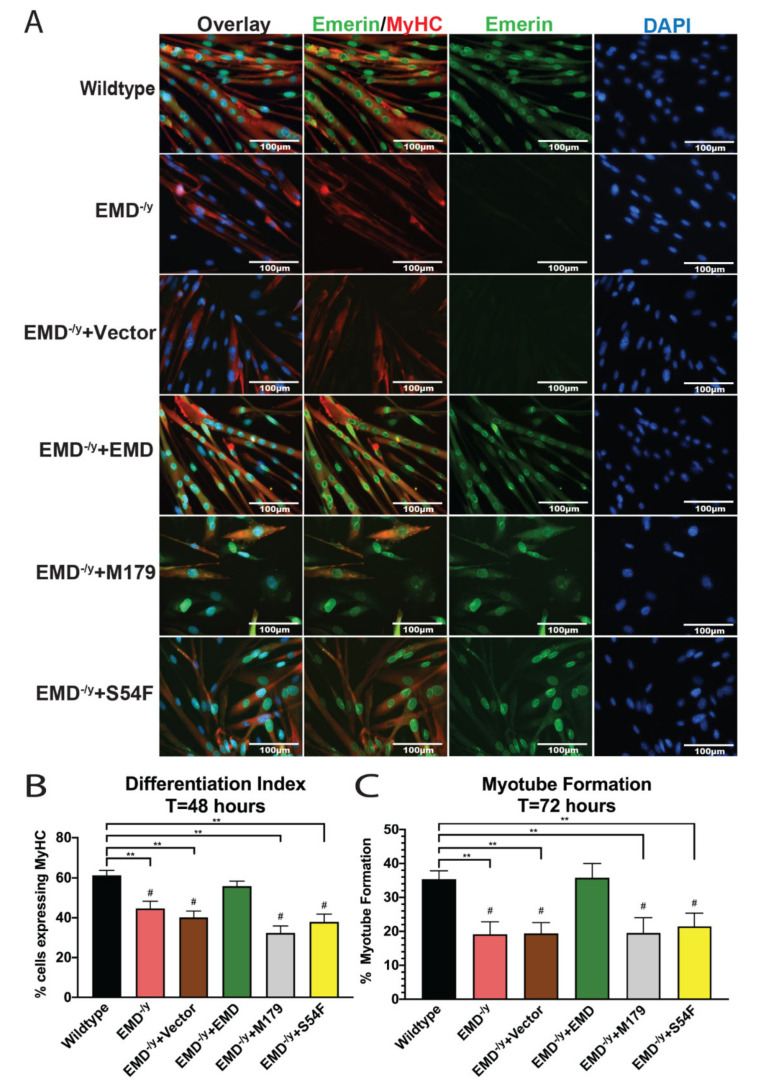
Emerin mutant myogenic progenitors M179 and S54F fail to form mature myotubes. (**A**) Immunofluorescence images at 72 h post differentiation induction. Scale bar: 100 µm. (**B**) The differentiation index at 48 h post differentiation induction. Emerin mutant progenitors M179 and S54F fail to rescue the differentiation index. (**C**) Myotube formation at 72 h after differentiation induction. Emerin mutant progenitors M179 and S54F fail to form mature myotubes after 72 h. **p < 0.02; #*p* < 0.05 in comparison to +EMD.

In total, 44.6% of EMD^−/y^ cells (Figure 3B; *p* < 0.02) and 40.2% of vector-alone cells (*p* < 0.02) expressed MyHC after 48 h of differentiation. In total, 61.3% of wildtype cells expressed MyHC. In total, 55.7% of +EMD cells expressed MyHC, demonstrating successful rescue of myoblast commitment. Expression of M179 and S54F in EMD^−/y^ progenitors failed to rescue MyHC expression and the differentiation index, with 32.4% and 37.9% expressing MyHC, respectively (Figure 3B; *p* < 0.02).

We next analyzed myotube formation, which was defined as the percentage of myotubes containing at least three nuclei and expressing MyHC. Myotube formation was impaired in EMD^−/y^ and control cells, as only 19.2% of EMD^−/y^ cells and 19.4% of control cells (Figure 3A,C; *p* < 0.02) formed mature myotubes compared to 35.4% of wildtype cells. Wildtype emerin expression rescued myotube formation, as 35.8% of +EMD cells formed myotubes. Both M179 and S54F cells were significantly impaired in myotube formation, as only 19.5% of M179 cells and 21.5% of S54F cells fused to form mature myotubes (Figure 3A,C; *p* < 0.02).

### 3.3. Q133H and Δ95–99 Mutant Myogenic Progenitors Exhibit Impaired Differentiation

**Figure 4:**
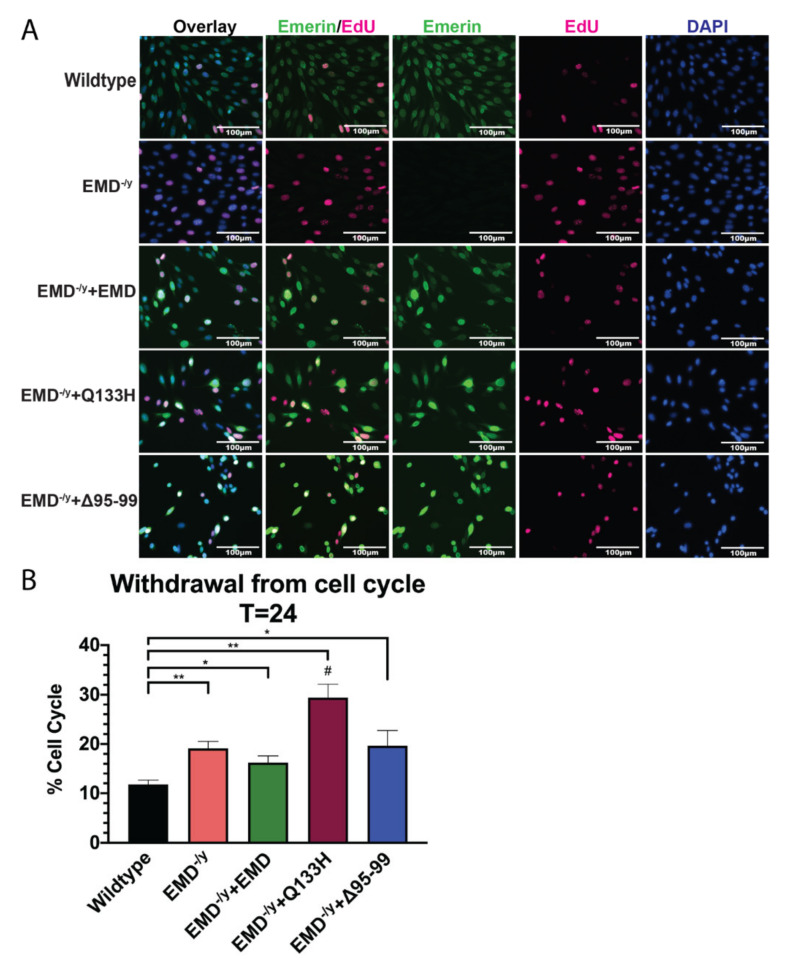
Cell cycle exit is impaired in EDMD-causing emerin mutant progenitors Δ95–99 and Q133H at 24 h post differentiation induction. (**A**) Immunofluorescence images at 24 h post differentiation induction. Scale bar: 100 µm. (**B**) Cell cycle withdrawal at 24 h after differentiation induction. Q133H and Δ95–99 emerin mutant myogenic progenitors fail to withdraw from the cell cycle. *p < 0.05, **p < 0.02; #*p* < 0.05 compared to +EMD.

Differentiation of EDMD-causing emerin mutant cell lines Q133H and Δ95–99 was assessed. Twenty-four hours after differentiation induction, EMD^−/y^ cells maintained a significantly higher percentage of cells in the cell cycle (19.11%; *p* < 0.02) compared to wildtype cells (11.73%; Figure 4A,B), consistent with previous results. Only 16.2% of +EMD cells incorporated EdU, demonstrating partial rescue of cell cycle withdrawal by emerin expression. In total, 29.4% of Q133H and 19.6% of Δ95–99 cells incorporated EdU.

**Figure 5:**
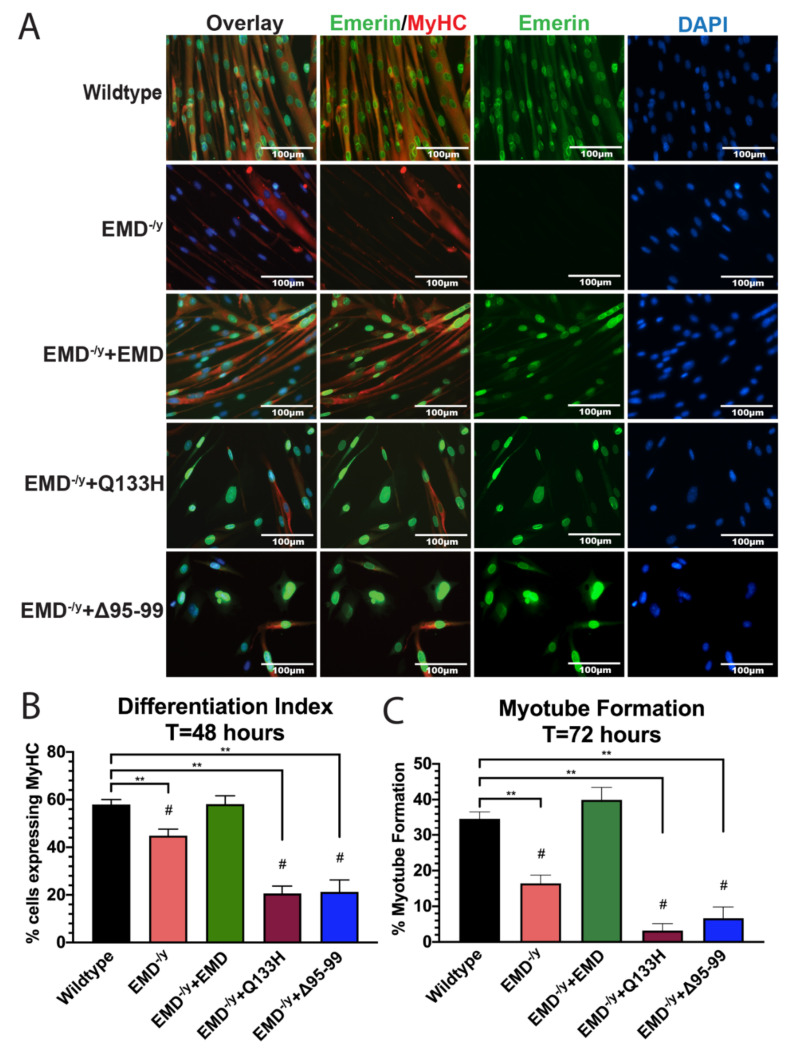
EDMD-causing emerin mutant myogenic progenitors Q133H and Δ95–99 display impaired myogenic differentiation. (**A**) Immunofluorescence images at 72 h post differentiation induction. Scale bar: 100 µm. (**B**) The differentiation index was calculated at 48 h after differentiation induction. Both Q133H and Δ95–99 showed decreased MyHC expression compared to wildtype cells. (**C**) Myotube formation was assessed at 72 h after differentiation induction. Both EDMD-causing emerin mutants completely failed to fuse and form myotubes after 72 h of differentiation. **p < 0.02; #*p* < 0.05 compared to +EMD.

As expected, the differentiation index of EMD^−/y^ progenitors (44.9%) was significantly lower than wildtype progenitors (58.0%) after 48 h of differentiation (Figure 5B; *p* < 0.02). In total, 58.1% of +EMD progenitors expressed MyHC after 48 h of differentiation. Both Q133H and Δ95–99 progenitors failed to commit to differentiation, as 20.6% of Q133H cells and 21.2% of Δ95–99 cells expressed MyHC (Figure 5B; *p* < 0.02).

After 72 h of differentiation, EMD^−/y^ cells failed to form myotubes (16.4%), as compared to wildtype cells (34.6%, Figure 5A,C; *p* < 0.02). In total, 39.9% of +EMD progenitors formed myotubes after 72 h. Only 3.2% of Q133H progenitors and 6.7% of Δ95–99 progenitors fused to form mature myotubes (Figure 5A,C; *p* < 0.02), demonstrating a massive failure in myotube formation and maturation in these EDMD-causing emerin mutants.

### 3.4. Identification of Pathways Shared Between all EDMD-Causing Emerin Mutants

RNA sequencing (RNAseq) was done on wildtype, EMD^−/y^, and each emerin mutant myogenic progenitor line throughout differentiation using Beijing Genomic Institute (BGI) Americas. Differentially expressed genes were identified by BGI by comparing all datasets to each other. The first comparison compared the changes in transcript expression at each day of differentiation for each EDMD-causing emerin mutant or EMD^−/y^ myogenic progenitors to wildtype progenitors. Transcripts were considered to be significantly differentially expressed if the *q*-value <0.05. It was expected that the expression of a large number of differentially expressed transcripts would be seen during differentiation of each EDMD-causing mutant based on previous studies [34].

**Figure 6:**
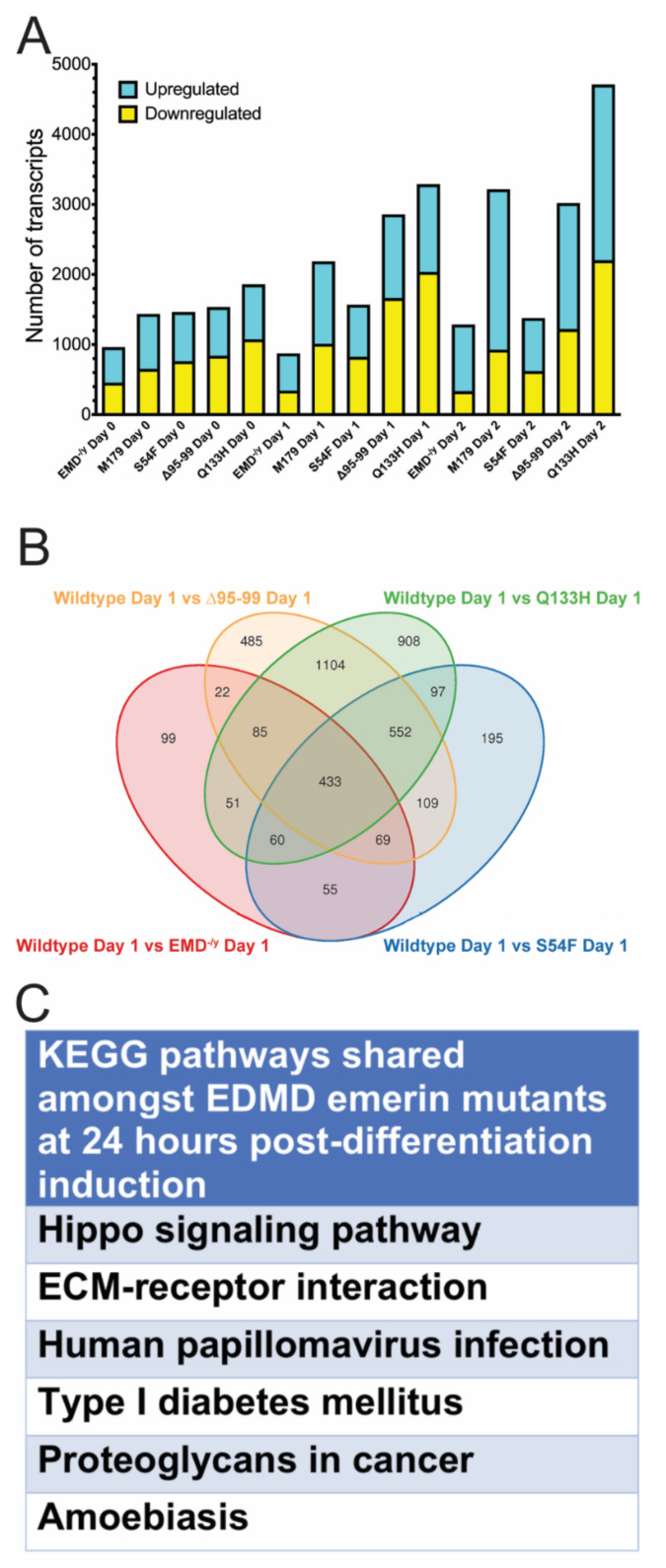
Significant changes in the transcriptome are seen during differentiation of EDMD mutant myogenic progenitors. (**A**) Differentially expressed transcripts were identified by comparing wildtype progenitors to EMD^−/y^ myogenic progenitors or each EDMD mutant myogenic progenitor at each differentiation day. **Blue**—upregulated transcripts; **Yellow**—downregulated transcripts. (**B**) Venn diagrams illustrating differentially expressed genes shared between one or more EDMD mutant progenitor one day after differentiation induction. (**C**) KEGG pathway analysis showing pathways enriched in EDMD emerin mutants, but not present in +EMD progenitors one day after differentiation induction.

In total, 956 differentially expressed genes (456 down, 508 up) were identified in EMD^−/y^ progenitors plated at high-density for differentiation (day 0, Figure 6A). S54F, Δ95–99, and Q133H myogenic progenitors differentially expressed 1468 genes (764 down, 702 up), 1536 genes (842 down, 694 up), and 1859 genes (1076 down, 783 up), respectively, when plated at high density (day 0, Figure 6A). There were 1338 genes (654 down, 784 up) differentially expressed in M179 progenitors upon plating at high density (Figure 6A). In total, 874 differentially expressed genes (344 down, 530 up) were identified in EMD^−/y^ progenitors one day after differentiation induction, which is when progenitors normally withdraw from the cell cycle to commit to differentiation (day 1, Figure 6A). S54F, Δ95–99, and Q133H myogenic progenitors differentially expressed 1570 genes (826 down, 744 up), 2859 genes (1666 down, 1193 up), and 3290 genes (2038 down, 1252 up), respectively (day 1, Figure 6A). There were 2188 (1011 down, 1177 up) differentially expressed genes in M179 progenitors at day 1. In total, 1286 differentially expressed genes (336 down, 950 up) were identified in EMD^−/y^ progenitors two days after differentiation induction, which is when myoblasts normally commit to form myotubes (day 2, Figure 6A). S54F, Δ95–99, and Q133H myogenic progenitors differentially expressed 1380 genes (623 down, 757 up), 3021 genes (1223 down, 1798 up), and 4714 genes (2207 down, 2507 up), respectively (day 2, Figure 6A). There were 3217 (930 down, 2287 up) differentially expressed genes in M179 progenitors at day 2.

EMD^−/y^, S54F, Δ95–99, Q133H, and M179 differentially expressed 795 genes (311 down, 484 up), 1111 genes (480 down, 631 up), 1464 genes (676 down, 688 up), 1287 genes (684 down, 603 up), and 1647 genes (925 down, 722 up) when actively proliferating, respectively (Figure S5). EMD^−/y^, S54F, Δ95–99, Q133H, and M179 differentially expressed 972 genes (321 down, 651 up), 1279 genes (573 down, 706 up), 2441 genes (1227 down, 1214 up), 3792 genes (1891 down, 1901 up), and 2526 genes (717 down, 1809 up) in mature myotubes, respectively (Figure S5; day 3).

We predicted that comparing the differentially expressed genes in all the EDMD-causing emerin mutants and EMD^−/y^ myogenic progenitors to wildtype progenitors would identify genes and pathways implicated in the impaired differentiation seen in EDMD. These analyses were focused first on differentiation day 1, as this timepoint is when wildtype progenitors exit the cell cycle to commit to becoming myotubes. In total, 433 genes were differentially expressed in each EDMD-causing emerin mutant cell lines and EMD^−/y^ cells, compared to wildtype cells (Figure 6B). KEGG pathway analysis was done to identify pathways shared amongst all differentiating EDMD-causing emerin mutants, but lacking in +EMD progenitors. Shared pathways were predicted to be important for the EDMD disease mechanism. Pathways enriched for these genes included Hippo signaling, ECM–receptor interactions, human papillomavirus infection, type I diabetes mellitus, proteoglycans in cancer, and amoebiasis (Figure 6C).

**Figure 7:**
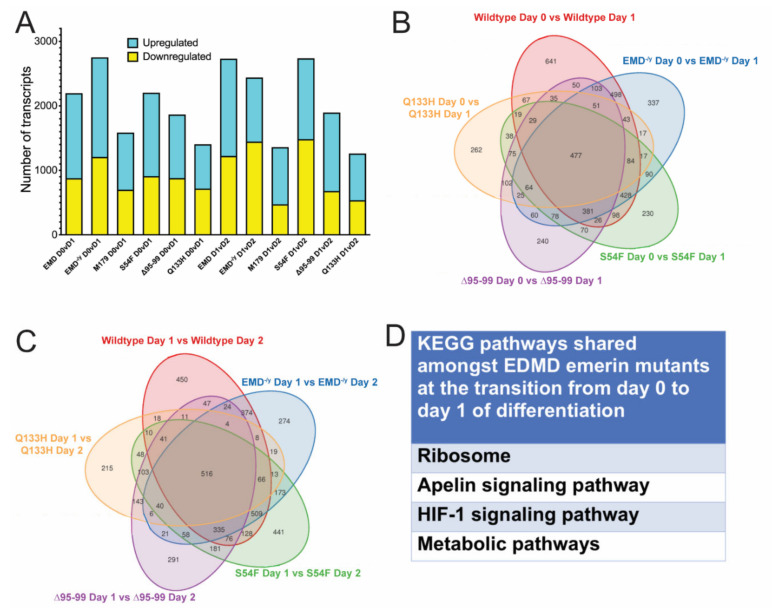
Significant changes in the transcriptome are seen at each daily transition during differentiation of EDMD mutant myogenic progenitors. (**A**) Differentially expressed transcripts were identified by comparing differentiation day 1 to differentiation day 0, or differentiation day 2 to differentiation day 1 in +EMD cells, EMD^−/y^ cells, or each EDMD mutant myogenic progenitor at each transition. **Blue**—upregulated transcripts; **Yellow**—downregulated transcripts. (**B**,**C**) Venn diagrams illustrating differentially expressed genes shared between wildtype, EMD^−/y^, and one or more EDMD mutant myogenic progenitors at the day 0 to day 1 transition, B, or day 1 to day 2 transition, C. (**D**) KEGG pathway analysis showing pathways enriched in EDMD emerin mutants, but not present in +EMD progenitors at the day 0 to day 1 transition.

A more powerful analysis, which we have used previously [34], was to compare transcripts differentially expressed between wildtype progenitors and either EMD^−/y^ or each EDMD-causing emerin mutant progenitors at important differentiation transitions. Differentially expressed genes between each day and the preceding day were determined for either differentiating +EMD, EMD^−/y^, or each EDMD-causing emerin mutant progenitor line. This analysis was anticipated to identify genes involved in the inability of EMD^−/y^ and EDMD-mutant progenitors to properly differentiate. In total, 2196 transcripts were differentially expressed upon withdrawal of +EMD progenitors from the cell cycle (day 0 to day 1; Figure 7A). The transition to committed myocytes (day 1 to day 2) caused 2731 genes to be differentially expressed in +EMD cells. In total, 1293 transcripts were differentially expressed upon plating +EMD progenitors at high-density for differentiation (prolif. to day 0; Figure S6), and 1804 genes were differentially expressed during myotube formation and maturation (day 2 to day 3; Figure S6). All differentially expressed transcripts had *q*-value <0.05.

Differentially expressed genes during each transition were determined for EMD^−/y^ myogenic progenitors and each EDMD-causing emerin mutant line. 2753 transcripts were differentially expressed upon the transition of EMD^−/y^ myogenic progenitors from differentiation day 0 to day 1, which is when cell cycle withdrawal should occur. The transition to myocyte commitment caused 2440 transcripts to be differentially expressed in EMD^−/y^ cells (Figure 7A; day 1 to day 2). In total, 985 transcripts were differentially expressed upon plating EMD^−/y^ myogenic progenitors at high-density (Figure S6; prolif. to day 0). 694 genes were differentially expressed during myotube formation and maturation (day 2 to day 3, Figure S6). In total, 2204 transcripts were differentially expressed upon the transition of S54F myogenic progenitors from differentiation day 0 to day 1. The transition to myocyte commitment caused 2738 transcripts to be differentially expressed in S54F cells (Figure 7A; day 1 to day 2). In total, 1046 transcripts were differentially expressed upon plating S54F myogenic progenitors at high-density (Figure S6; prolif. to day 0). 1057 genes were differentially expressed during myotube formation and maturation (day 2 to day 3, Figure S6). In total, 1866 transcripts were differentially expressed upon the transition of Δ95–99 myogenic progenitors from differentiation day 0 to day 1. The transition to myocyte commitment caused 1897 transcripts to be differentially expressed in Δ95–99 cells (Figure 7A; day 1 to day 2). In total, 1203 transcripts were differentially expressed upon plating Δ95–99 myogenic progenitors at high-density (Figure S6; prolif. to day 0). In total, 1324 genes were differentially expressed during myotube formation and maturation (day 2 to day 3, Figure S6). In total, 1405 transcripts were differentially expressed upon the transition of Q133H myogenic progenitors from differentiation day 0 to day 1. The transition to myocyte commitment caused 1261 transcripts to be differentially expressed in Q133H cells (Figure 7A; day 1 to day 2). In total, 930 transcripts were differentially expressed upon plating Q133H myogenic progenitors at high-density (Figure S6; prolif. to day 0). 1179 genes were differentially expressed during myotube formation and maturation (day 2 to day 3, Figure S6). All differentially expressed transcripts had *q*-value <0.05.

Transcripts differentially expressed at each daily transition in differentiating EMD^−/y^ progenitors or each EDMD-causing mutant progenitor line were then compared to wildtype progenitors at each transition. This identified transcripts differentially expressed only in differentiating wildtype or EMD^−/y^ progenitors, or wildtype progenitors and each EDMD emerin mutant progenitor line, or any comparison between all the samples at each transition point (Figure 7B). For example, transcripts that are differentially expressed during wildtype differentiation between day 0 and day 1 of differentiation were compared to the differentially expressed transcripts seen during the transition from differentiation day 0 to day 1 in EMD^−/y^ cells, S54F cells, Δ95–99 cells, and Q133H cells. This comparison yields genes uniquely differentially expressed in wildtype cells, EMD^−/y^ cells, S54F cells, Δ95–99 cells, and Q133H cells during the transition from day 0 to day 1 of differentiation (Figure 7B). This approach also allows for comparisons between all possible combinations to identify differentially expressed genes shared between certain EDMD-causing emerin mutants or uniquely absent from certain EDMD mutant cell lines.

A large number of transcripts were differentially expressed between day 0 and day 2 of differentiation in wildtype, EMD^−/y^, and each EDMD-causing emerin mutant progenitor line (Figure 7B,C), as expected [34]; this is when the differentiation program is initiated and commitment occurs. Of the 2753 transcripts differentially expressed during EMD^−/y^ differentiation days 0 and 1, 2065 transcripts were also altered during wildtype differentiation, and 688 transcripts were uniquely altered in EMD EMD^−/y^ cells (Figure 7B; *q*-value <0.05). In total, 2440 transcripts showed altered expression in EMD^−/y^ cells during the transition from day 1 to day 2 of myogenic differentiation (Figure 7C; *q*-value < 0.05), with 1836 transcripts also altered during wildtype differentiation and 604 transcripts uniquely altered in EMD^−/y^ cells. Of the 2204 transcripts differentially expressed during S54F differentiation days 0 and 1, 1542 transcripts were also altered during wildtype differentiation and 662 transcripts were uniquely altered in S54F cells (Figure 7B; *q*-value <0.05). In total, 2738 transcripts showed altered expression in S54F cells during the transition from day 1 to day 2 of myogenic differentiation (Figure 7C; *q*-value <0.05), with 1681 transcripts also altered during wildtype differentiation and 1057 transcripts uniquely altered in S54F cells. Of the 1866 transcripts differentially expressed during Δ95–99 differentiation days 0 and 1, 1152 transcripts were also altered during wildtype differentiation and 714 transcripts were uniquely altered in Δ95–99 cells (Figure 7B; *q*-value <0.05). In total, 1897 transcripts showed altered expression in Δ95–99 cells during the transition from day 1 to day 2 of myogenic differentiation (Figure 7C; *q*-value <0.05), with 1054 transcripts also altered during wildtype differentiation and 843 transcripts uniquely altered in Δ95–99 cells. Of the 1405 transcripts differentially expressed during Q133H differentiation days 0 and 1, 805 transcripts were also altered during wildtype differentiation and 600 transcripts were uniquely altered in Q133H cells (Figure 7B; *q*-value <0.05). In total, 1261 transcripts showed altered expression in Q133H cells during the transition from day 1 to day 2 of myogenic differentiation (Figure 7C; *q*-value <0.05), with 674 transcripts also altered during wildtype differentiation and 587 transcripts uniquely altered in Q133H cells. To identify genes implicated in the impaired differentiation of EDMD, transcripts differentially expressed between wildtype progenitors and each EDMD-causing emerin mutant progenitor line and EMD^−/y^ cells were compared during these transitions. This identified 64 transcripts and 40 transcripts differentially expressed in each EDMD mutant and EMD^−/y^ cells during transitions day 0 to day 1 and day 1 to day 2, respectively (Figure 7B,C).

KEGG pathway analysis identified KEGG pathways enriched in the EDMD-causing emerin mutants, but absent from +EMD cells at each differentiation transition. These were compared to identify those pathways that were shared only amongst the differentiating EDMD-causing mutant myogenic progenitors (Figure 7D). The shared pathways are predicted to be important for the EDMD disease mechanism. Pathways enriched among all EDMD mutants during differentiation transition day 0 to day 1 were ribosome, apelin signaling pathway, HIF-1 signaling, and metabolic pathways (Figure 7D). The Rap1 signaling pathway and tight junction pathway were both enriched in two of the three EDMD emerin mutant lines, with Rap1 enriched in Δ95–99 and S54F, and tight junction enriched in S54F and Q133H. Pathways enriched amongst all EDMD mutants during differentiation transition day 1 to day 2 were the p53 signaling pathway and homologous recombination. The PI3K-Akt signaling pathway was enriched in Δ95–99 and S54F cell lines.

## 4. Discussion

In this study, we utilized lentiviral delivery to express EDMD-causing emerin mutants (S54F, Δ95–99, and Q133H) in an EMD^−/y^ myogenic progenitor background. We next differentiated wildtype, EMD^−/y^, and +EMD progenitors to validate our experimental approach. The differentiation index and myotube formation were successfully rescued to wildtype levels in +EMD progenitors, while +EMD progenitors failed to rescue cell cycle withdrawal after 24 h of differentiation. This indicates emerin may not be important for regulating cell cycle exit, but rather regulate later differentiation transitions. This is similar to the effects we previously saw upon treatment with pharmacological activators of HDAC3 activity [30], histone acetylase inhibitors [38], and inhibitors of ERK activity [30]. Collectively, the results presented here, combined with our earlier studies [30,34,35,36,38], strongly support emerin regulating transcriptional reprograming events early in differentiation to impact myoblast commitment and myotube formation through its functional interaction with HDAC3.

The genome undergoes dynamic reorganization during stem cell differentiation and development to regulate the coordinated temporal expression of differentiation genes [42,43,44]. It has been reported that active genes localize towards the nuclear interior whereas repressed genes preferentially localize at the nuclear periphery [43,45,46]. Activation, commitment, and differentiation of myogenic progenitors are controlled by various epigenetic mechanisms, which include histone modifications, RNA-associated silencing, and DNA methylation [42,44,47]. The importance of histone acetylation and deacetylation in the regulation of myogenesis has been established [48,49,50,51]. Although global histone acetylation decreases progressively during myogenesis, a subset of genes, such as MyoD target genes, show increased histone acetylation upon differentiation [48,52,53] concomitant with their expression. To date, class 1 and class 2 HDACs have been repeatedly reported to function in different phases of myogenesis. The results presented here, combined with our previous work [15,30,34,36,38], support an important role for emerin in activating HDAC3 activity to decrease histone acetylation during myogenic differentiation. We propose this interaction mediates the establishment, recruitment, and maintenance of repressed chromatin at the nuclear lamina [30], and that this reorganization of the genome at the nuclear lamina during differentiation induction is required for transcriptional reprogramming. 

HDAC3 is the only reported binding partner that is altered in all four “special” EDMD-causing emerin mutations (Q133H, P183H, S54F, and Δ95–99) [15]. This highlights the direct binding and functional interaction between HDAC3 and emerin may be important for the EDMD disease mechanism. Our studies revealed EDMD-causing emerin mutants Δ95–99 and Q133H exhibit impaired differentiation. After 24 h of differentiation induction, Δ95–99 and Q133H cells fail to commit to differentiation; they fail to express MyHC, a terminal marker for differentiation; and fail to form mature myotubes. Notably, both of these mutants fail to bind HDAC3 [15]. EDMD-causing emerin mutant S54F also failed to rescue cell cycle withdrawal, the differentiation index, and myotube formation. Surprisingly M179, an emerin mutant not linked to EDMD, also failed to rescue cell cycle withdrawal, the differentiation index and myotube formation. The M179 mutant contains two alanine-substitutions at position 179 and 180 for leucine and serine. Although these mutants also disrupt binding to other partners [13,15,16,17,18,19,20,37,54], only failure to bind HDAC3 is shared between all of the mutants tested here. We propose perturbation of this interaction in the EDMD-causing emerin mutants results in failure in genomic reorganization and failure to transcribe genes required for myoblast commitment and myotube formation. Supporting this hypothesis, inhibition of HDAC3 catalytic activity by RGFP966 blocked MyHC expression and myotube formation in both differentiating wildtype and EMD^−/y^ myogenic progenitors [30]. It will be important to determine how emerin regulates genomic organization via HDAC3 regulation to affect transcriptional reprogramming. Understanding how specific molecular pathways are impacted by disruption of the functional interaction of emerin with HDAC3 will also be important for elucidating the mechanism(s) underlying the impaired differentiation seen in EDMD-causing mutants.

Emerin mutants M179, S54F, and Q133H all showed an increased number of cells cycling 24 h after differentiation induction compared to EMD^−/y^ progenitors. Δ95–99 myogenic progenitors were similar to EMD^−/y^ cells in their cell cycle exit. We predict this failure of M179, S54F, and Q133H progenitors to exit the cell cycle is caused by binding to germ cell-less (GCL). GCL is a transcriptional repressor that binds the DP3 subunit of the E2F–DP3 heterodimer to inactivate E2F–DP3-mediated transcription [55] and inhibit entry into S-phase. GCL binds directly to emerin via Regulator Binding Domains (RBD) 1 and RBD-2 in emerin [18]. We predict GCL and HDAC3 compete for binding emerin based upon their overlapping binding sites. There is precedent for this, as GCL and BAF compete for binding to emerin [18].

Entry into the cell cycle is regulated by the G1/S checkpoint, which is also called the restriction point [56,57]. Passage of the restriction point requires activation of E2F–DP, which transcribes many cell cycle genes [58,59,60,61,62,63]. Rb binding to the E2F–DP heterodimer is known to convert E2F–DP from a potent transcription activator to a potent transcription repressor [64,65,66]. Only hypophosphorylated Rb binds to the E2F–DP heterodimer [67,68,69,70]. Rb-bound E2F–DP then recruits HDACs to repress the chromatin to which it is bound.

In our model, increased hyperphosphorylated Rb in EMD^−/y^ mice is the primary driving force for passage through the restriction point in EMD^−/y^ mice, as first described by Melcon and colleagues [27]. Here, increased hyperphosphorylated Rb caused increased E2F-mediated transcription that persisted after differentiation induction. This delay eventually resolved after a few days and myotubes eventually formed in vitro and in vivo. These events were predicted to be a compensatory mechanism allowing skeletal muscle formation and regeneration to occur in EMD^−/y^ mice, which have surprisingly mild phenotypes [27,28]. In our model, emerin can bind HDAC3 and GCL in EMD^−/y^ progenitors expressing wildtype emerin. GCL binding sites at the nuclear envelope would not be saturated because HDAC3 and GCL compete with each other for emerin binding. In this model there would be insufficient recruitment of GCL to alter the dynamics of E2F–DP-mediated transcription activation in the presence of high levels of Rb and hyperphosphorylated Rb in EMD^−/y^ cells. Δ95–99 fails to bind GCL and HDAC3, so this mutant behaves just like the EMD^−/y^ parental cell line. In M179-, S54F-, or Q133H-expressing EMD^−/y^ progenitors, these mutants retain their ability to bind GCL but fail to bind HDAC3. Thus, emerin can bind more GCL, thereby releasing more GCL from E2F–DP to further increase the expression of E2F–DP-regulated genes that allow for passage through the restriction point.

We predict HDAC3 also plays a role in repressing transcription of cell cycle genes, but it is unclear whether it acts with Rb or GCL to repress E2F-regulated transcripts in cycling cells or if it acts to stably repress E2F-regulated chromatin in permanently arrested cells, like myotubes. Our data would suggest the latter. Thus, it will be important to test whether HDAC3 association with emerin plays a role in the compensatory mechanisms predicted to help stabilize repressed chromatin containing E2F-regulated genes, and if this is required for permanent cell cycle arrest. It will also be important to determine the dynamics of GCL and HDAC3 competition for emerin binding and how this affects passage through the restriction point. Whether expression of wildtype emerin in EMD^−/y^ cells alters Rb phosphorylation dynamics will also need to be tested.

Emerin mutants Q133H and Δ95–99 had less MyHC expression after 48 h and formed fewer myotubes after 72 h of differentiation than EMD^−/y^ cells. S54F and M179 were similar to EMD^−/y^ cells in differentiation index and myotube formation. Both S54F and M179 bind normally to lamin A, whereas Q133H and Δ95–99 disrupt binding to lamin A [71]. We speculate that failure to bind lamin A in Q133H and Δ95–99 mutants contributes to their decreased differentiation. One possibility is loss of lamin A binding to emerin may impair nuclear structure [72,73], which may lead to increased senescence or death and subsequently less myotubes. Failure of lamin A to bind Q133H and Δ95–99 mutants may also disrupt stabilization of repressed chromatin at the nuclear periphery [74,75], which may affect the expression of genes important for myoblast commitment and myotube formation. Q133H and Δ95–99 also fail to self-assemble into curvilinear structures in vitro [26], suggesting this failure could contribute to a more severe phenotype either due to an unstable emerin conformation or due to failure to bind an unidentified protein that regulates myogenic differentiation. Another hypothesis would involve compensatory mechanisms via other inner nuclear membrane proteins such as Lap2β [27,29] and Lap1 [76]. Lap2β is similar to emerin in its LEM, transmembrane, and nucleoplasmic domains [1,71]. Both these proteins show some functional redundancy with emerin and their expression levels are upregulated in the absence of emerin [76,77]. It is possible that Lap1 and Lap2β are compensating for loss of some functions in EMD^−/y^ cells that may involve binding to and repressing an unknown partner at the inner nuclear membrane. In this functional redundancy model, expression of the Q133H and Δ95–99 mutants in the EMD^−/y^ cells specifically alters binding to this unknown partner and more of the partner is now active to negatively repress myogenic differentiation. Alternatively, release of this partner from emerin now allows it to bind to Lap2β or Lap1 and displaces other binding partners on these proteins that negatively impact myogenic differentiation.

The transcriptomic analysis of the EDMD-causing emerin mutants uncovered pathways implicated in myogenic differentiation. Members of the Hippo family play vital roles in skeletal muscle development [78], organ size control [79], and tissue regeneration [80] in mammalian cells. Two transcriptional cofactors within the Hippo pathway, Yap (*Yap1*) and Taz (*Wwtr1*), primarily regulate gene expression by binding Tead1–4 transcription factors. Together Yap, Taz, and Teads form the core of the Hippo signal transduction network, which includes the Hippo kinase cascade, comprising kinases Mst1 (*Stk4*), Mst2 (*Stk3*), Lats1, and Lats2 [80,81,82]. Yap and Taz were shown to regulate myoblast proliferation and terminal differentiation in regenerating myoblasts in vivo and in satellite cells derived from isolated mouse muscle fibers [83,84,85]. Increased Yap and Taz expression is associated with myogenic progenitor proliferation [83,85,86]. Sustained Yap upregulation is sufficient to inhibit the terminal differentiation program vital for myocyte fusion and formation of myofibers [85,86]. Interestingly, sustained Taz upregulation enhances terminal differentiation of myoblasts in vitro and in vivo [87,88,89]. Yap was recently shown to contribute to the pathogenesis in muscular dystrophy, as increased nuclear localization of Yap is seen in lamin-A-related congenital muscular dystrophy (L-CMD) [90]. Our analysis also revealed significant upregulation of Yap expression and activity along with upregulation of several other pathways’ components in the EDMD-causing emerin mutants, suggesting there is increased Yap localization in the nucleus that promotes proliferation activity and inhibits the differentiation program [83]. Therefore, one can speculate that persistent activation of Yap in the EDMD-causing emerin mutants has negative effects on myogenic differentiation.

There is increasing evidence suggesting extracellular matrix (ECM) components are essential signaling mediators in the satellite cell niche, both for the maintenance of satellite cell identity and regulation of its activation [91,92]. Heparan sulfate proteoglycans syndecan-3 and syndecan-4 regulate satellite cell activation and satellite cell self-renewal is impaired in syndecan-4 knockout mice [93,94]. The ECM provides stimuli for muscle cell development, which is independent of the expression of muscle-specific transcription factors Myf-5 and MyoD [93,95,96]. During differentiation, heparan sulfate proteoglycans interact with multiple growth factors in the muscle basal lamina, including insulin-like growth factor (IGF), fibroblast growth factor (FGF), hepatocyte growth factor (HGF), and transforming growth factor beta (TGF-β), which are well established regulators of satellite cell proliferation and differentiation [92,97,98]. The dynamic expression of heparan sulfate proteoglycans during skeletal muscle regeneration and differentiation likely reflects different functions for these complex molecules [99,100]. EDMD-causing emerin mutants showed significantly decreased expression for collagen, laminin, von Willebrand factor, thrombospondin 1, reelin, and vitronectin, revealing alterations in the ECM–receptor pathway needed for regulation of cellular events vital for muscle growth, repair and to mediate cell–cell adhesion during skeletal muscle regeneration. Laminin is essential for key processes that occur during differentiation, where it enhances myoblast proliferation, migration, and alignment involved in myotube formation; absence of laminin impairs myogenic differentiation [101]. In EDMD, this would be predicted to cause aberrant myogenic regeneration due to the altered response of myogenic progenitors to the available growth factors needed for progenitor activation and myotube formation [102,103,104].

Apelin is locally produced by skeletal muscle fibers in response to exercise and acts locally to improve muscle metabolism and function [105]. Apelin is expressed, secreted, and responsive to exercise-activated signaling pathways in cultured human primary myotubes [105]. Apelin also stimulates energy expenditure by increasing vascular mass and mitochondrial biogenesis [106]. Several lines of evidence indicate mitochondrial activity and function are linked to myogenic differentiation [107,108,109,110,111]. Mitochondrial activity is increased when myoblasts differentiate into myotubes [112,113,114]. During myogenesis, there is a metabolic shift from glycolysis to oxidative phosphorylation as the major energy source, suggesting this metabolic shift may be a crucial event that regulates cell differentiation [115,116,117,118,119]. Our analysis revealed that EDMD-causing emerin mutants show alterations in apelin and associated metabolic pathways, suggesting that these pathways may be responsible for a metabolic shift that impairs myogenesis due to diminished energy utilization. The function of Hypoxia-inducible factor 1α (HIF1α) in myoblasts has been controversial. Two groups revealed hypoxia-induced HIF1α accumulation inhibited myoblast differentiation [120,121], whereas another study revealed that HIF1α knockdown inhibited myoblast differentiation in normoxic conditions [122]. A recent study reported that HIF1α inhibited ischemia-induced muscle regeneration by inhibiting Wnt signaling [121]. Collectively, the function of HIFs in muscle stem cells in vivo is not well understood. Whether Apelin or HIF-1 signaling are important in the impaired myogenic differentiation seen in EDMD remains to be determined.

## 5. Conclusions

These studies show, for the first time, that emerin mutations found in EDMD patients cause impaired myogenic differentiation. The impaired differentiation is likely caused by loss of HDAC3 binding, since these mutants share in their failure to bind HDAC3. The use of these individual mutants allowed for a robust approach to identifying pathways implicated in the impaired differentiation in EDMD. Using this approach, we narrowed the implicated pathways to four to six pathways. Future studies to independently test each of these pathways will be needed to confirm their involvement in the impaired differentiation seen in EDMD.

## Supplementary Materials

The following are available online at https://www.mdpi.com/2073-4409/9/6/1463/s1, Figure S1: Cell cycle withdrawal 48 h after differentiation induction of M179 and S54F mutant myogenic progenitors, Figure S2: Cell cycle withdrawal 72 hours after differentiation induction of M179 and S54F myogenic progenitors, Figure S3: Cell cycle withdrawal 48 h after differentiation induction of EDMD mutant progenitor lines Q133H and Δ95–99, Figure S4: Cell cycle withdrawal 72 h after differentiation induction of EDMD mutant progenitor lines Q133H and Δ95–99, Figure S5: Significant changes in the transcriptome are seen during differentiation of EDMD mutant myogenic progenitors, Figure S6: Significant changes in the transcriptome are seen at each daily transition during differentiation of EDMD mutant myogenic progenitors.

## References

[1] Bione S., Maestrini E., Rivella S., Mancini M., Regis S., Romeo G., Toniolo D. (1994). Identification of a novel X-linked gene responsible for Emery-Dreifuss muscular dystrophy. Nat. Genet..

[2] Méndez-López I., Worman H.J. (2012). Inner nuclear membrane proteins: Impact on human disease. Chromosoma.

[3] Vlcek S., Foisner R. (2007). Lamins and lamin-associated proteins in aging and disease. Curr. Opin. Cell Boil..

[4] Worman H.J. (2011). Nuclear lamins and laminopathies. J. Pathol..

[5] Yates J.R., Wehnert M. (1999). The Emery-Dreifuss Muscular Dystrophy Mutation Database. Neuromuscul. Disord..

[6] Manilal S., Man N.T., Sewry C.A., Morris G.E. (1996). The Emery-Dreifuss muscular dystrophy protein, emerin, is a nuclear membrane protein. Hum. Mol. Genet..

[7] Manilal S., Manila S., Recan D., Sewry C.A., Hoeltzenbein M., Llense S., Leturcq F., Deburgrave N., Barbot J.-C., Man N.T. (1998). Mutations in Emery-Dreifuss muscular dystrophy and their effects on emerin protein expression. Hum. Mol. Genet..

[8] Manilal S., Sewry C.A., Man N.T., Muntoni F., Morris G.E. (1997). Diagnosis of X-linked Emery-Dreifuss muscular dystrophy by protein analysis of leucocytes and skin with monoclonal antibodies. Neuromuscul. Disord..

[9] Nagano A., Koga R., Ogawa M., Kurano Y., Kawada J., Okada R., Hayashi Y.K., Tsukahara T., Arahata K. (1996). Emerin deficiency at the nuclear membrane in patients with Emery-Dreif uss muscular dystrophy. Nat. Genet..

[10] Ellis A.J., Craxton M., Yates J.R., Kendrick-Jones J. (1998). Aberrant intracellular targeting and cell cycle-dependent phosphorylation of emerin contribute to the Emery-Dreifuss muscular dystrophy phenotype. J. Cell Sci..

[11] Mora M., Cartegni L., Di Blasi C., Barresi R., Bione S., Di Barletta M.R., Morandi L., Merlini L., Nigro V., Politano L. (1997). X-linked emery-dreifuss muscular dystrophy can be diagnosed from skin biopsy or blood sample. Ann. Neurol..

[12] Yates J.R., Bagshaw J., Aksmanovic V.M., Coomber E., McMahon R., Whittaker J.L., Morrison P.J., Kendrick-Jones J., Ellis A.J. (1999). Genotype-phenotype analysis in X-linked Emery-Dreifuss muscular dystrophy and identification of a missense mutation associated with a milder phenotype. Neuromuscul. Disord..

[13] Berk J.M., Simon D.N., Jenkins-Houk C.R., Westerbeck J.W., Grønning-Wang L.M., Carlson C.R., Wilson K.L. (2014). The molecular basis of emerin-emerin and emerin-BAF interactions. J. Cell Sci..

[14] Herrada I., Bourgeois B., Samson C., Buendia B., Worman H.J., Zinn-Justin S. (2016). Purification and Structural Analysis of LEM-Domain Proteins. Methods Enzymol..

[15] Demmerle J., Koch A.J., Holaska J.M. (2012). The Nuclear Envelope Protein Emerin Binds Directly to Histone Deacetylase 3 (HDAC3) and Activates HDAC3 Activity. J. Boil. Chem..

[16] Mislow J.M., Holaska J.M., Kim M.S., Lee K.K., Segura-Totten M., Wilson K.L., McNally E.M. (2002). Nesprin-1α self-associates and binds directly to emerin and lamin A in vitro. FEBS Lett..

[17] Haraguchi T., Holaska J.M., Yamane M., Koujin T., Hashiguchi N., Mori C., Wilson K.L., Hiraoka Y. (2004). Emerin binding to Btf, a death-promoting transcriptional repressor, is disrupted by a missense mutation that causes Emery-Dreifuss muscular dystrophy. JBIC J. Boil. Inorg. Chem..

[18] Holaska J.M., Lee K.K., Kowalski A.K., Wilson K.L. (2002). Transcriptional Repressor Germ Cell-less (GCL) and Barrier to Autointegration Factor (BAF) Compete for Binding to Emerin in Vitro. J. Boil. Chem..

[19] Holaska J.M., Rais-Bahrami S., Wilson K.L. (2006). Lmo7 is an emerin-binding protein that regulates the transcription of emerin and many other muscle-relevant genes. Hum. Mol. Genet..

[20] Holaska J.M., Kowalski A.K., Wilson K.L. (2004). Emerin Caps the Pointed End of Actin Filaments: Evidence for an Actin Cortical Network at the Nuclear Inner Membrane. PLoS Boil..

[21] Markiewicz E., Tilgner K., Barker N., van de Wetering M., Clevers H., Dorobek M., Hausmanowa-Petrusewicz I., Ramaekers F., Broers J., Blankesteijn W.M. (2006). The inner nuclear membrane protein emerin regulates beta-catenin activity by restricting its accumulation in the nucleus. EMBO J..

[22] Salpingidou G., Smertenko A., Hausmanowa-Petrucewicz I., Hussey P.J., Hutchison C. (2007). A novel role for the nuclear membrane protein emerin in association of the centrosome to the outer nuclear membrane. J. Cell Boil..

[23] Fairley E.A., Kendrick-Jones J., Ellis J.A. (1999). The Emery-Dreifuss muscular dystrophy phenotype arises from aberrant targeting and binding of emerin at the inner nuclear membrane. J. Cell Sci..

[24] Ellis J.A., Yates J.R., Kendrick-Jones J., Brown C.A. (1999). Changes at P183 of emerin weaken its protein-protein interactions resulting in X-linked Emery-Dreifuss muscular dystrophy. Qual. Life Res..

[25] Holt I., Clements L., Manilal S., Morris G.E. (2001). How does a g993t mutation in the emerin gene cause Emery-Dreifuss muscular dystrophy?. Biochem. Biophys. Res. Commun..

[26] Herrada I., Samson C., Velours C., Renault L., Östlund C., Chervy P., Puchkov D., Worman H.J., Buendia B., Zinn-Justin S. (2015). Muscular Dystrophy Mutations Impair the Nuclear Envelope Emerin Self-assembly Properties. ACS Chem. Boil..

[27] Melcon G., Kozlov S., Cutler D.A., Sullivan T., Hernandez L., Zhao P., Mitchell S., Nader G., Bakay M., Rottman J.N. (2006). Loss of emerin at the nuclear envelope disrupts the Rb1/E2F and MyoD pathways during muscle regeneration. Hum. Mol. Genet..

[28] Ozawa R., Hayashi Y.K., Ogawa M., Kurokawa R., Matsumoto H., Noguchi S., Nonaka I., Nishino I. (2006). Emerin-Lacking Mice Show Minimal Motor and Cardiac Dysfunctions with Nuclear-Associated Vacuoles. Am. J. Pathol..

[29] Bakay M., Wang Z., Melcon G., Schiltz L., Xuan J., Zhao P., Sartorelli V., Seo J., Pegoraro E., Angelini C. (2006). Nuclear envelope dystrophies show a transcriptional fingerprint suggesting disruption of Rb–MyoD pathways in muscle regeneration. Brain.

[30] Collins C.M., Ellis J.A., Holaska J.M. (2017). MAPK signaling pathways and HDAC3 activity are disrupted during differentiation of emerin-null myogenic progenitor cells. Dis. Model. Mech..

[31] Frock R.L., Kudlow B.A., Evans A.M., Jameson S.A., Hauschka S.D., Kennedy B.K. (2006). Lamin A/C and emerin are critical for skeletal muscle satellite cell differentiation. Genome Res..

[32] Huber M.D., Guan T., Gerace L. (2009). Overlapping Functions of Nuclear Envelope Proteins NET25 (Lem2) and Emerin in Regulation of Extracellular Signal-Regulated Kinase Signaling in Myoblast Differentiation. Mol. Cell. Boil..

[33] Dedeic Z., Cetera M., Cohen T., Holaska J.M. (2011). Emerin inhibits Lmo7 binding to the Pax3 and MyoD promoters and expression of myoblast proliferation genes. J. Cell Sci..

[34] Iyer A., Koch A.J., Holaska J.M. (2017). Expression Profiling of Differentiating Emerin-Null Myogenic Progenitor Identifies Molecular Pathways Implicated in Their Impaired Differentiation. Cells.

[35] Koch A.J., Holaska J.M. (2012). Loss of Emerin Alters Myogenic Signaling and miRNA Expression in Mouse Myogenic Progenitors. PLoS ONE.

[36] Demmerle J., Koch A.J., Holaska J.M. (2013). Emerin and histone deacetylase 3 (HDAC3) cooperatively regulate expression and nuclear positions of MyoD, Myf5, and Pax7 genes during myogenesis. Chromosom. Res..

[37] Holaska J.M., Wilson K.L. (2007). An Emerin “Proteome”: Purification of Distinct Emerin-Containing Complexes from HeLa Cells Suggests Molecular Basis for Diverse Roles Including Gene Regulation, mRNA Splicing, Signaling, Mechanosensing, and Nuclear Architecture. Biochemistry.

[38] Bossone K.A., Ellis J.A., Holaska J.M. (2020). Histone acetyltransferase inhibition rescues differentiation of emerin-deficient myogenic progenitors. Muscle Nerve.

[39] Toyoshima K., Vogt P.K. (1969). Enhancement and inhibition of avian sarcoma viruses by polycations and polyanions. Virology.

[40] Coelen R.J., Jose D.G., May J.T. (1983). The effect of hexadimethrine bromide (polybrene) on the infection of the primate retroviruses SSV 1/SSAV 1 and BaEV. Arch. Virol..

[41] Davis H.E., Morgan J.R., Yarmush M.L. (2002). Polybrene increases retrovirus gene transfer efficiency by enhancing receptor-independent virus adsorption on target cell membranes. Biophys. Chem..

[42] Sousa-Victor P., Muñoz-Canoves P., Perdiguero E. (2011). Regulation of skeletal muscle stem cells through epigenetic mechanisms. Toxicol. Mech. Methods.

[43] Reddy K., Zullo J.M., Bertolino E., Singh H. (2008). Transcriptional repression mediated by repositioning of genes to the nuclear lamina. Nature.

[44] Juan A.H., Wang S., Ko K.D., Zare H., Tsai P.-F., Feng X., Vivanco K.O., Ascoli A.M., Gutierrez-Cruz G., Krebs J. (2016). Roles of H3K27me2 and H3K27me3 Examined During Fate Specification of Embryonic Stem Cells. Cell Rep..

[45] Kind J., Pagie L., Ortabozkoyun H., Boyle S., De Vries S.S., Janssen H., Amendola M., Nolen L.D., Bickmore W.A., Van Steensel B. (2013). Single-Cell Dynamics of Genome-Nuclear Lamina Interactions. Cell.

[46] Zullo J.M., Demarco I.A., Pique-Regi R., Gaffney D.J., Epstein C.B., Spooner C.J., Luperchio T.R., Bernstein B.E., Pritchard J.K., Reddy K. (2012). DNA Sequence-Dependent Compartmentalization and Silencing of Chromatin at the Nuclear Lamina. Cell.

[47] Faralli H., Wang C., Nakka K., Benyoucef A., Sebastian S., Zhuang L., Chu A., Palii C.G., Liu C., Camellato B. (2016). UTX demethylase activity is required for satellite cell-mediated muscle regeneration. J. Clin. Investig..

[48] Puri P.L., Iezzi S., Stiegler P., Chen T.-T., Schiltz R., Muscat G., Giordano A., Kedes L., Wang J.Y., Sartorelli V. (2001). Class I histone deacetylases sequentially interact with MyoD and pRb during skeletal myogenesis. Mol. Cell.

[49] Caretti G., Di Padova M., Micales B., Lyons G.E., Sartorelli V. (2004). The Polycomb Ezh2 methyltransferase regulates muscle gene expression and skeletal muscle differentiation. Genome Res..

[50] Mal A., Harter M.L. (2003). MyoD is functionally linked to the silencing of a muscle-specific regulatory gene prior to skeletal myogenesis. Proc. Natl. Acad. Sci. USA.

[51] Ohkawa Y., Marfella C.G., Imbalzano A.N. (2006). Skeletal muscle specification by myogenin and Mef2D via the SWI/SNF ATPase Brg1. EMBO J..

[52] Cao Y., Yao Z., Sarkar D., Lawrence M., Sanchez G.J., Parker M.H., MacQuarrie K., Davison J., Morgan M., Ruzzo W.L. (2010). Genome-wide MyoD Binding in Skeletal Muscle Cells: A Potential for Broad Cellular Reprogramming. Dev. Cell.

[53] Jin W., Peng J., Jiang S. (2016). The epigenetic regulation of embryonic myogenesis and adult muscle regeneration by histone methylation modification. Biochem. Biophys. Rep..

[54] Berk J.M., Tifft E.K., Wilson K.L. (2013). The nuclear envelope LEM-domain protein emerin. Nucleus.

[55] De La Luna S., Allen K., Mason S.L., La Thangue N.B. (1999). Integration of a growth-suppressing BTB/POZ domain protein with the DP component of the E2F transcription factor. EMBO J..

[56] Pardee A. (1989). G1 events and regulation of cell proliferation. Science.

[57] Pardee A.B. (1974). A Restriction Point for Control of Normal Animal Cell Proliferation. Proc. Natl. Acad. Sci. USA.

[58] DeGregori J., Kowalik T., Nevins J.R. (1995). Cellular targets for activation by the E2F1 transcription factor include DNA synthesis- and G1/S-regulatory genes. Mol. Cell. Biol..

[59] Asano M., Nevins J.R., Wharton R.P. (1996). Ectopic E2F expression induces S phase and apoptosis in Drosophila imaginal discs. Genes Dev..

[60] Dyson N.J. (1998). The regulation of E2F by pRB-family proteins. Genome Res..

[61] Lukas J., Petersen O.B., Holm K., Bartek J., Helin K. (1996). Deregulated expression of E2F family members induces S-phase entry and overcomes p16INK4A-mediated growth suppression. Mol. Cell. Boil..

[62] Chen H.-Z., Tsai S., Leone G. (2009). Emerging roles of E2Fs in cancer: An exit from cell cycle control. Nat. Rev. Cancer.

[63] Johnson D.G. (1998). Role of E2F in cell cycle control and cancer. Front. Biosci..

[64] Pollard T.D., Pollard T.D., Earnshaw W.C., Lippincott-Schwartz J., Johnson G.T., Earnshaw W.C. (2017). Cell Biology.

[65] A Henley S., Dick F.A. (2012). The retinoblastoma family of proteins and their regulatory functions in the mammalian cell division cycle. Cell Div..

[66] Uchida C. (2016). Roles of pRB in the Regulation of Nucleosome and Chromatin Structures. BioMed Res. Int..

[67] Bandara L.R., Adamczewski J.P., Hunt T., La Thangue N.B. (1991). Cyclin A and the retinoblastoma gene product complex with a common transcription factor. Nature.

[68] Chellappan S.P., Hiebert S., Mudryj M., Horowitz J.M., Nevins J.R. (1991). The E2F transcription factor is a cellular target for the RB protein. Cell.

[69] Chittenden T., Livingston D.M., Kaelin W.G. (1991). The T/E1A-binding domain of the retinoblastoma product can interact selectively with a sequence-specific DNA-binding protein. Cell.

[70] Hiebert S.W., Horowitz J.M., Chellappan S.P., Nevins J.R. (1992). The interaction of RB with E2F coincides with an inhibition of the transcriptional activity of E2F. Genes Dev..

[71] Lee K.K., Haraguchi T., Lee R.S., Koujin T., Hiraoka Y., Wilson K.L. (2001). Distinct functional domains in emerin bind lamin A and DNA-bridging protein BAF. J. Cell Sci..

[72] Lammerding J., Schulze P.C., Takahashi T., Kozlov S., Sullivan T., Kamm R.D., Stewart C.L., Lee R.T. (2004). Lamin A/C deficiency causes defective nuclear mechanics and mechanotransduction. J. Clin. Investig..

[73] Sullivan T., Escalante-Alcalde D., Bhatt H., Anver M., Bhat N., Nagashima K., Stewart C.L., Burke B. (1999). Loss of a-Type Lamin Expression Compromises Nuclear Envelope Integrity Leading to Muscular Dystrophy. J. Cell Boil..

[74] Dechat T., Pfleghaar K., Sengupta K., Shimi T., Shumaker D.K., Solimando L., Goldman R.D. (2008). Nuclear lamins: Major factors in the structural organization and function of the nucleus and chromatin. Genome Res..

[75] Dittmer T., Misteli T. (2011). The lamin protein family. Genome Boil..

[76] Shin J.-Y., Méndez-López I., Wang Y., Hays A.P., Tanji K., Lefkowitch J.H., Schulze P.C., Worman H.J., Dauer W.T. (2013). Lamina-associated polypeptide-1 interacts with the muscular dystrophy protein emerin and is essential for skeletal muscle maintenance. Dev. Cell.

[77] Koch A.J., Holaska J.M. (2013). Emerin in health and disease. Semin. Cell Dev. Boil..

[78] Leach J., Heallen T., Zhang M., Rahmani M., Morikawa Y., Hill M.C., Segura A., Willerson J.T., Martin J.F. (2017). Hippo pathway deficiency reverses systolic heart failure after infarction. Nature.

[79] Camargo F.D., Gokhale S., Johnnidis J.B., Fu D., Bell G.W., Jaenisch R., Brummelkamp T.R. (2007). YAP1 increases organ size and expands undifferentiated progenitor cells. Curr. Biol..

[80] Wackerhage H., Del Re M.P., Judson R.N., Sudol M., Sadoshima J. (2014). The Hippo signal transduction network in skeletal and cardiac muscle. Sci. Signal..

[81] Hansen C., Moroishi T., Guan K.-L. (2015). YAP and TAZ: A nexus for Hippo signaling and beyond. Trends Cell Boil..

[82] Harvey K.F., Tapon N. (2007). The Salvador–Warts–Hippo pathway—An emerging tumour-suppressor network. Nat. Rev. Cancer.

[83] Judson R.N., Tremblay A.M., Knopp P., White R., Urcia R., De Bari C., Zammit P.S., Camargo F.D., Wackerhage H. (2012). The Hippo pathway member Yap plays a key role in influencing fate decisions in muscle satellite cells. J. Cell Sci..

[84] Park G.H., Jeong H., Jeong M.-G., Jang E.J., Bae M.A., Lee Y.-L., Kim N.J., Hong J.-H., Hwang E.S. (2014). Novel TAZ modulators enhance myogenic differentiation and muscle regeneration. Br. J. Pharmacol..

[85] Tremblay A.M., Missiaglia E., Galli G.G., Hettmer S., Urcia R., Carrara M., Judson R.N., Thway K., Nadal G., Selfe J.L. (2014). The Hippo Transducer YAP1 Transforms Activated Satellite Cells and Is a Potent Effector of Embryonal Rhabdomyosarcoma Formation. Cancer Cell.

[86] Watt K.I., Judson R., Medlow P., Reid K., Kurth T.B., Burniston J.G., Ratkevicius A., De Bari C., Wackerhage H. (2010). Yap is a novel regulator of C2C12 myogenesis. Biochem. Biophys. Res. Commun..

[87] Jeong H., Bae S., An S.Y., Byun M.R., Hwang J.-H., Yaffe M.B., Hong J.-H., Hwang E.S. (2010). TAZ as a novel enhancer of MyoD-mediated myogenic differentiation. FASEB J..

[88] Mohamed A., Sun C., De Mello V., Selfe J.L., Missiaglia E., Shipley J., Murray I.G., Zammit P.S., Wackerhage H. (2016). The Hippo effector TAZ (WWTR1) transforms myoblasts and TAZ abundance is associated with reduced survival in embryonal rhabdomyosarcoma. J. Pathol..

[89] Sun C., De Mello V., Mohamed A., Quiroga H.P.O., Garcia-Munoz A., Al Bloshi A., Tremblay A.M., Von Kriegsheim A., Collie-Duguid E., Vargesson N. (2017). Common and Distinctive Functions of the Hippo Effectors Taz and Yap in Skeletal Muscle Stem Cell Function. STEM CELLS.

[90] Owens D.J., Fischer M., Jabre S., Moog S., Mamchaoui K., Butler-Browne G., Coirault C. (2020). Lamin Mutations Cause Increased YAP Nuclear Entry in Muscle Stem Cells. Cells.

[91] Hay E.D. (2013). Cell Biology of Extracellular Matrix.

[92] Thomas K., Engler A.J., Meyer G.A. (2014). Extracellular matrix regulation in the muscle satellite cell niche. Connect. Tissue Res..

[93] Cornelison D., Filla M.S., Stanley H.M., Rapraeger A.C., Olwin B.B. (2001). Syndecan-3 and Syndecan-4 Specifically Mark Skeletal Muscle Satellite Cells and Are Implicated in Satellite Cell Maintenance and Muscle Regeneration. Dev. Boil..

[94] Cornelison D., Wilcox-Adelman S.A., Goetinck P.F., Rauvala H., Rapraeger A.C., Olwin B.B. (2004). Essential and separable roles for Syndecan-3 and Syndecan-4 in skeletal muscle development and regeneration. Genome Res..

[95] Osses N., Brandan E. (2002). ECM is required for skeletal muscle differentiation independently of muscle regulatory factor expression. Am. J. Physiol. Physiol..

[96] Osses N., Casar J.C., Brandan E. (2009). Inhibition of extracellular matrix assembly induces the expression of osteogenic markers in skeletal muscle cells by a BMP-2 independent mechanism. BMC Cell Boil..

[97] Allen R.E., Boxhorn L.K. (1989). Regulation of skeletal muscle satellite cell proliferation and differentiation by transforming growth factor-beta, insulin-like growth factor I, and fibroblast growth factor. J. Cell. Physiol..

[98] Gutieérrez J., Brandan E. (2010). A Novel Mechanism of Sequestering Fibroblast Growth Factor 2 by Glypican in Lipid Rafts, Allowing Skeletal Muscle Differentiation. Mol. Cell. Boil..

[99] Jenniskens G.J., Hafmans T., Veerkamp J.H., Van Kuppevelt T.H. (2002). Spatiotemporal distribution of heparan sulfate epitopes during myogenesis and synaptogenesis: A study in developing mouse intercostal muscle. Dev. Dyn..

[100] Liu X., Nestor K., McFarland D.C., Velleman S.G. (2002). Developmental expression of skeletal muscle heparan sulfate proteoglycans in turkeys with different growth rates. Poult. Sci..

[101] Sanes J.R. (2003). The Basement Membrane/Basal Lamina of Skeletal Muscle. J. Boil. Chem..

[102] Miura T., Kishioka Y., Wakamatsu J., Hattori A., Nishimura T. (2010). Interaction between myostatin and extracellular matrix components. Anim. Sci. J..

[103] Yasaka N., Wakamatsu J., Suzuki K., Kishioka Y., Nishimura T. (2013). Laminin binds to myostatin and attenuates its signaling. Anim. Sci. J..

[104] Dodson M.V. (2010). Skeletal Muscle Stem Cells from Animals I. Basic Cell Biology. Int. J. Boil. Sci..

[105] Besse-Patin A., Montastier E., Vinel C., Castan-Laurell I., Louche K., Dray C., Daviaud D., Mir L., Marques M.-A., Thalamas C. (2013). Effect of endurance training on skeletal muscle myokine expression in obese men: Identification of apelin as a novel myokine. Int. J. Obes..

[106] Yamamoto T., Habata Y., Matsumoto Y., Yasuhara Y., Hashimoto T., Hamajyo H., Anayama H., Fujii R., Fuse H., Shintani Y. (2011). Apelin-transgenic mice exhibit a resistance against diet-induced obesity by increasing vascular mass and mitochondrial biogenesis in skeletal muscle. Biochim. Biophys. Acta (BBA) Gen. Subj..

[107] Hamai N., Nakamura M., Asano A. (1997). Inhibition of Mitochondrial Protein Synthesis Impaired C2C12 Myoblast Differentiation. Cell Struct. Funct..

[108] Herzberg N.H., Middelkoop E., Adorf M., Dekker H.L., Van Galen M.J., Berg M.V.D., Bolhuis A.P., Bogert C.V.D. (1993). Mitochondria in cultured human muscle cells depleted of mitochondrial DNA. Eur. J. Cell Boil..

[109] Korohoda W., Pietrzkowski Z., Reiss K. (1993). Chloramphenicol, an inhibitor of mitochondrial protein synthesis, inhibits myoblast fusion and myotube differentiation. Folia Histochem. Cytobiol..

[110] Rochard P., Rodier A., Casas F., Cassar-Malek I., Marchal-Victorion S., Daury L., Wrutniak C., Cabello G. (2000). Mitochondrial activity is involved in the regulation of myoblast differentiation through myogenin expression and activity of myogenic factors. J. Boil. Chem..

[111] Seyer P., Grandemange S., Busson M., Carazo Á., Gamaléri F., Pessemesse L., Casas F., Cabello G., Wrutniak-Cabello C. (2006). Mitochondrial activity regulates myoblast differentiation by control of c-Myc expression. J. Cell. Physiol..

[112] Barbieri E., Battistelli M., Casadei L., Vallorani L., Piccoli G., Guescini M., Gioacchini A.M., Polidori E., Zeppa S., Ceccaroli P. (2011). Morphofunctional and Biochemical Approaches for Studying Mitochondrial Changes during Myoblasts Differentiation. J. Aging Res..

[113] Moyes C., Mathieu-Costello O.A., Tsuchiya N., Filburn C., Hansford R.G. (1997). Mitochondrial biogenesis during cellular differentiation. Am. J. Physiol. Content.

[114] Remels A.H.V., Langen R., Schrauwen P., Schaart G., Schols A., Gosker H.R. (2010). Regulation of mitochondrial biogenesis during myogenesis. Mol. Cell. Endocrinol..

[115] Chung S., Dzeja P.P., Faustino R.S., Perez-Terzic C., Behfar A., Terzic A. (2007). Mitochondrial oxidative metabolism is required for the cardiac differentiation of stem cells. Nat. Clin. Pr. Neurol..

[116] Chung S., Dzeja P.P., Faustino R.S., Terzic A. (2008). Developmental restructuring of the creatine kinase system integrates mitochondrial energetics with stem cell cardiogenesis. Ann. N. Y. Acad. Sci..

[117] Fink E., Fortin D., Serrurier B., Ventura-Clapier R., Bigard A. (2003). Recovery of contractile and metabolic phenotypes in regenerating slow muscle after notexin-induced or crush injury. J. Muscle Res. Cell Motil..

[118] Wagatsuma A., Kotake N., Yamada S. (2010). Muscle regeneration occurs to coincide with mitochondrial biogenesis. Mol. Cell. Biochem..

[119] Duguez S., Féasson L., Denis C., Freyssenet D. (2002). Mitochondrial biogenesis during skeletal muscle regeneration. Am. J. Physiol. Metab..

[120] Gustafsson M.V., Zheng X., Pereira T., Gradin K., Jin S., Lundkvist J., Ruas J.L., Poellinger L., Lendahl U., Bondesson M. (2005). Hypoxia Requires Notch Signaling to Maintain the Undifferentiated Cell State. Dev. Cell.

[121] Majmundar A.J., Lee D.S.M., Skuli N., Mesquita R.C., Kim M.N., Yodh A.G., Nguyen-McCarty M., Li B., Simon M.C. (2015). HIF modulation of Wnt signaling regulates skeletal myogenesis in vivo. J. Cell Sci..

[122] Ono Y., Sensui H., Sakamoto Y., Nagatomi R. (2006). Knockdown of hypoxia-inducible factor-1α by siRNA inhibits C2C12 myoblast differentiation. J. Cell. Biochem..

