# Supplementary material for: EDMD-Causing Emerin Mutant Myogenic Progenitors Exhibit Impaired Differentiation Using Similar Mechanisms
Source: Cells. 2020 Jun 15;9(6):1463. doi: 10.3390/cells9061463 (PMC7349064; doi:10.3390/cells9061463)
Supplement: Supplementary file 1 [file cells-09-01463-s001.pdf]

At 48 h after differentiation induction (Figure S1A,B), all wildtype cells withdrew from the cell cycle whereas EMD$^{-/y}$, vector only, +EMD and M179 cells displayed a significantly higher percentage of cells in the cell cycle. At 72 h post differentiation induction (Figure S2A,B), all cell lines withdrew from the cell cycle whereas EMD$^{-/y}$ ($p < 0.05$) and vector only cells ($p < 0.02$) were still significantly active in the cell cycle.

Withdrawal from cell cycle at 48 h after differentiation revealed EMD$^{-/y}$, +EMD, Q133H and Δ95-99 exhibited a significantly higher percentage of cells in the cell cycle ($p < 0.02$) compared to wildtype cells, which had no cells in the cell cycle (Figure S3A,B). At 72 h after differentiation, EMD$^{-/y}$ had a significant percentage of cells in the cell cycle ($p < 0.02$) whereas all other cell lines rescued cell cycle withdrawal (Figure S4A,B).

Changes in transcript expression at each day of differentiation for each EDMD-causing emerin mutant or EMD$^{-/y}$ myogenic progenitors compared to wildtype progenitors were determined in proliferating cells, cells plated at high density (day 0) and each day throughout differentiation until mature myotubes were formed (Figure S5). Differentially expressed genes during the transition from proliferative to plating at high density (PvD0) and each daily transition during myogenic differentiation were determined for EMD$^{-/y}$ myogenic progenitors and each EDMD-causing emerin mutant line (Figure S6). Transcripts were considered to be significantly differentially expressed if the q-value <0.05.

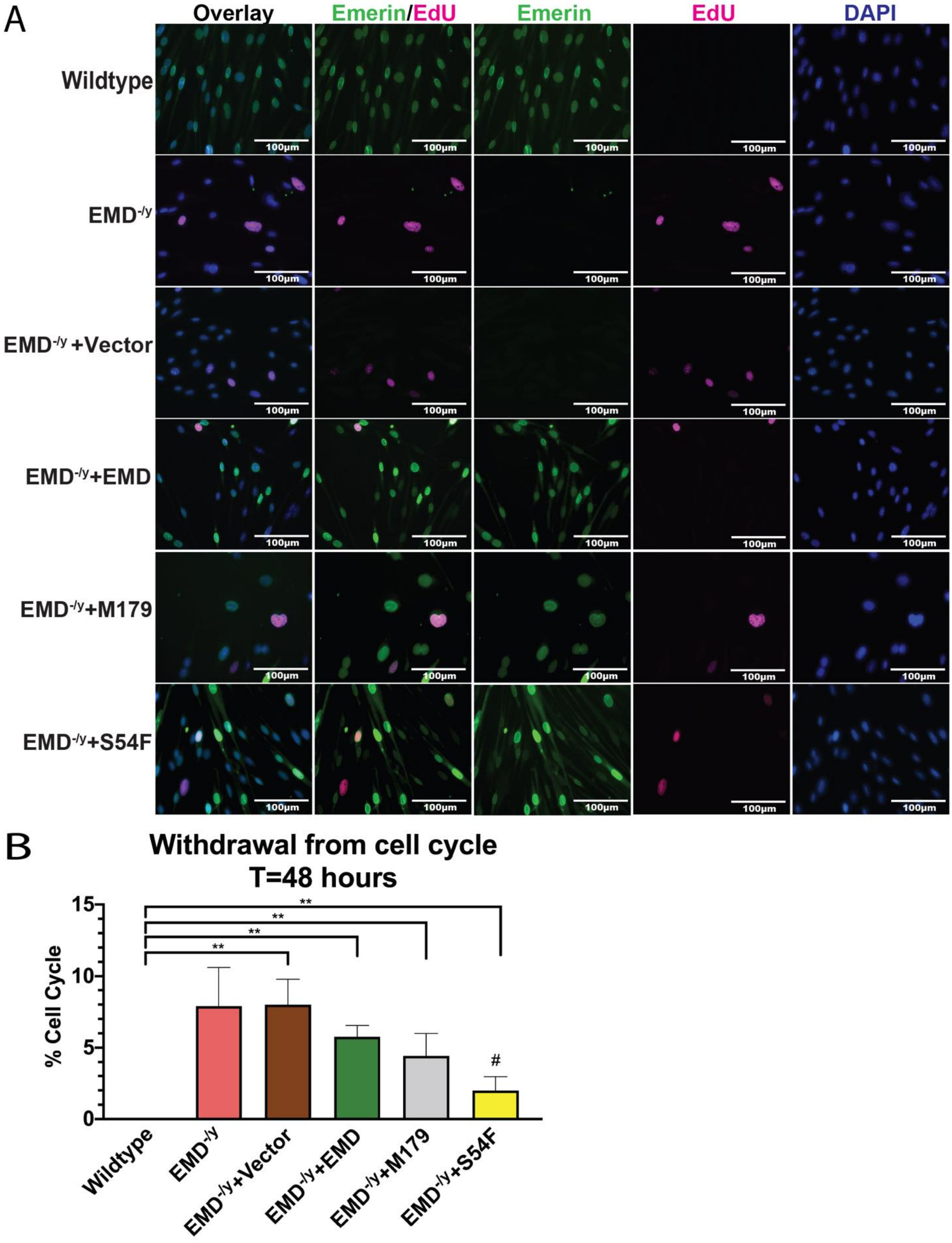

Figure S1. Cell cycle withdrawal 48 h after differentiation induction of M179 and S54F mutant myogenic progenitors. (**A**) Immunofluorescence images at 48 h post differentiation induction. Scale bar: 100 µm. (**B**) The percentage of cells in the cell cycle was calculated for each progenitor line 48 h after differentiation induction. No wildtype cells incorporated EdU, whereas EMD$^{-/y}$, vector alone, +EMD and M179 cell lines displayed a significant number of cells that incorporated EdU. *$p < 0.05$, **$p < 0.02$; #$p < 0.05$ in comparison to +EMD.

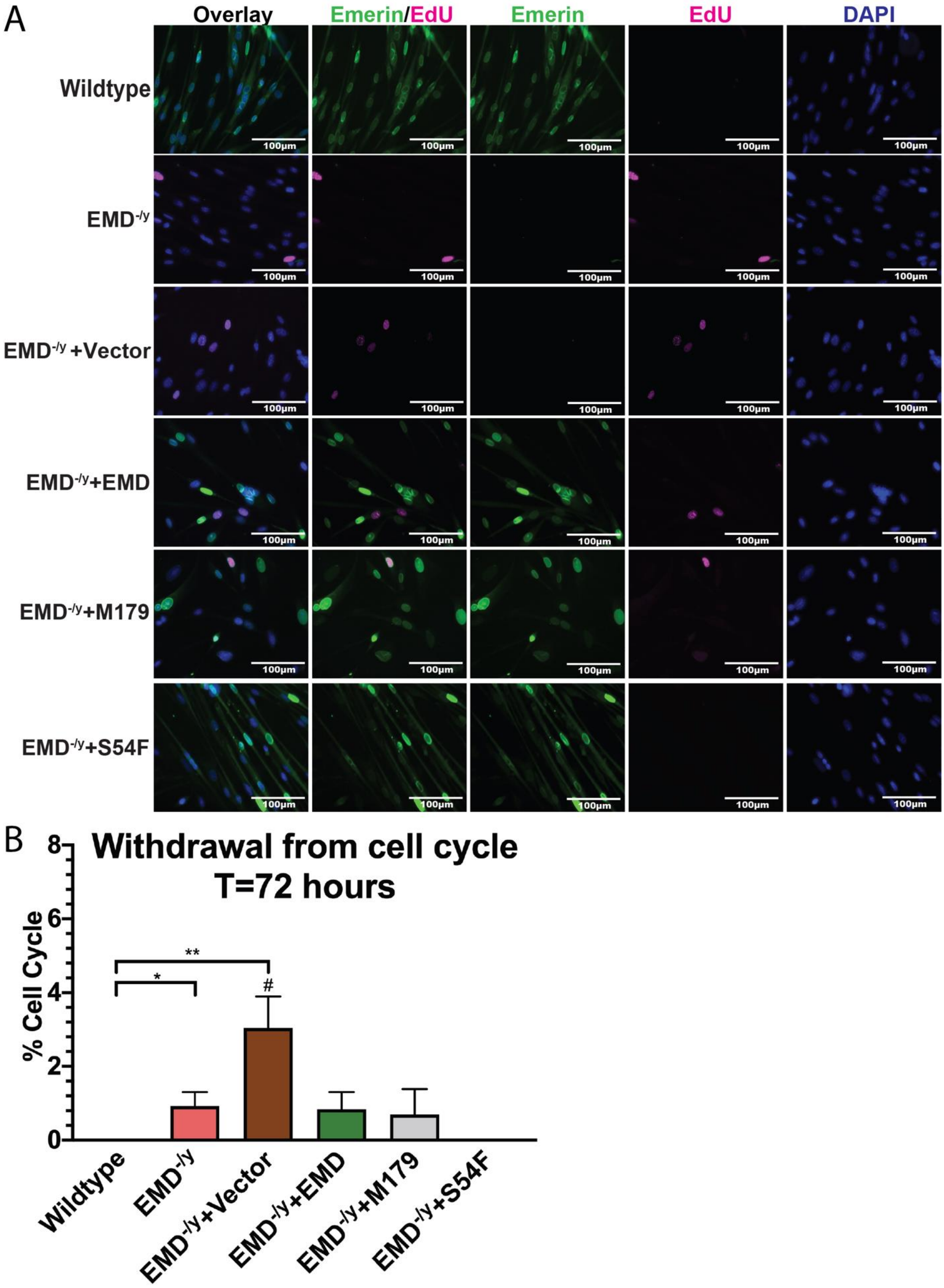

Figure S2. Cell cycle withdrawal 72 h after differentiation induction of M179 and S54F myogenic progenitors. (**A**) Immunofluorescence images at 72 h post differentiation induction. Scale bar: 100 µm. (**B**) The percentage of cells in the cell cycle was calculated for each progenitor line 72 h after differentiation induction. All cell lines except for EMD$^{-/y}$ and vector control withdrew from the cell cycle. *$p < 0.05$, **$p < 0.02$; #$p < 0.05$ in comparison to +EMD.

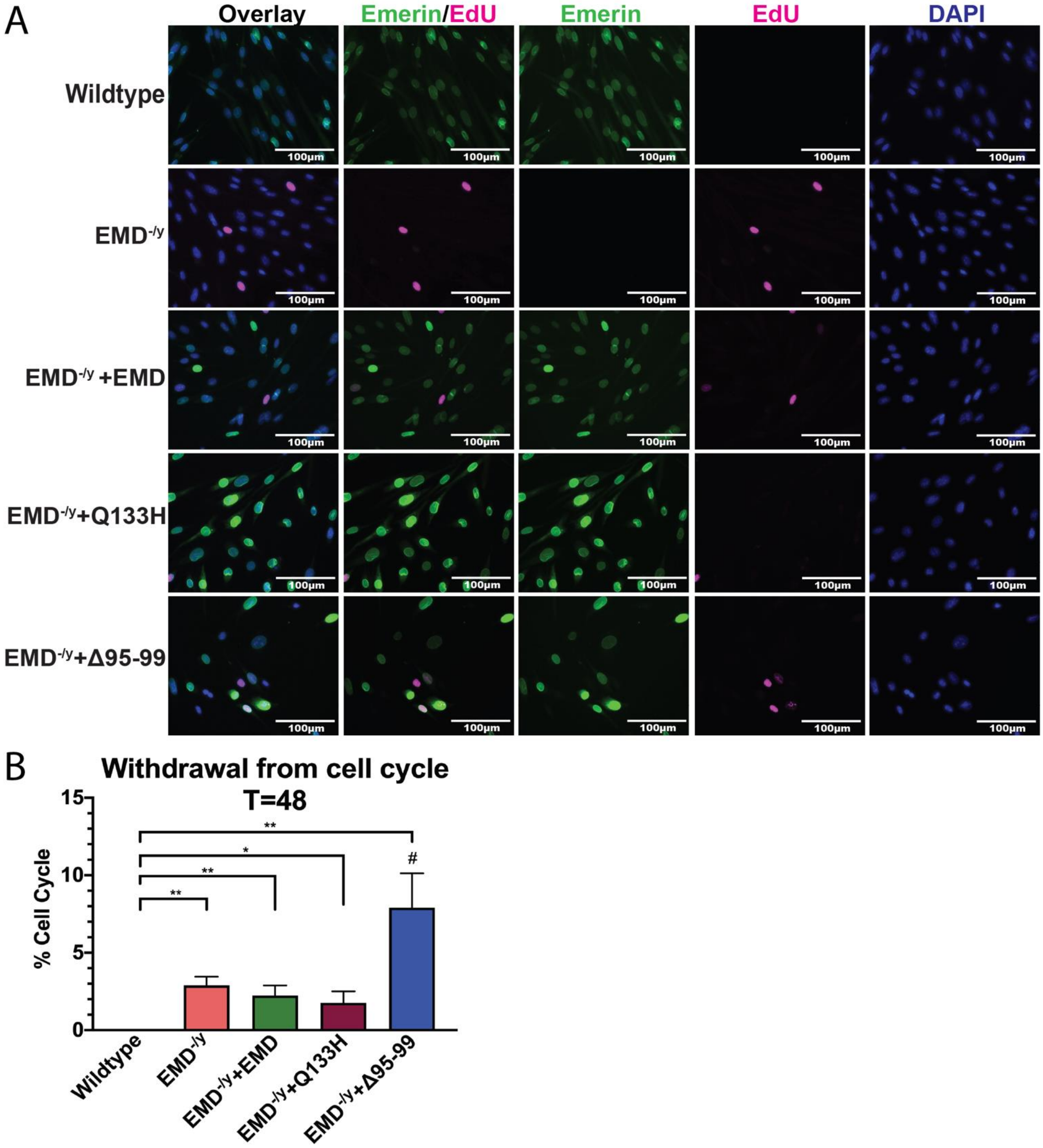

Figure S3. Cell cycle withdrawal 48 h after differentiation induction of EDMD mutant progenitor lines Q133H and Δ95-99. (**A**) Immunofluorescence images at 48 h after differentiation induction. Scale bar: 100 μm. (**B**) The percentage of cells in the cell cycle was calculated for each progenitor line 48 h after differentiation induction. EMD$^{-/y}$, +EMD, Q133H and Δ95–99 progenitors exhibited a significantly higher percentage of cells in the cell cycle compared to wildtype cells, which completely exited the cell cycle. *p<0.05, **p<0.02; #p<0.05 in comparison to +EMD.

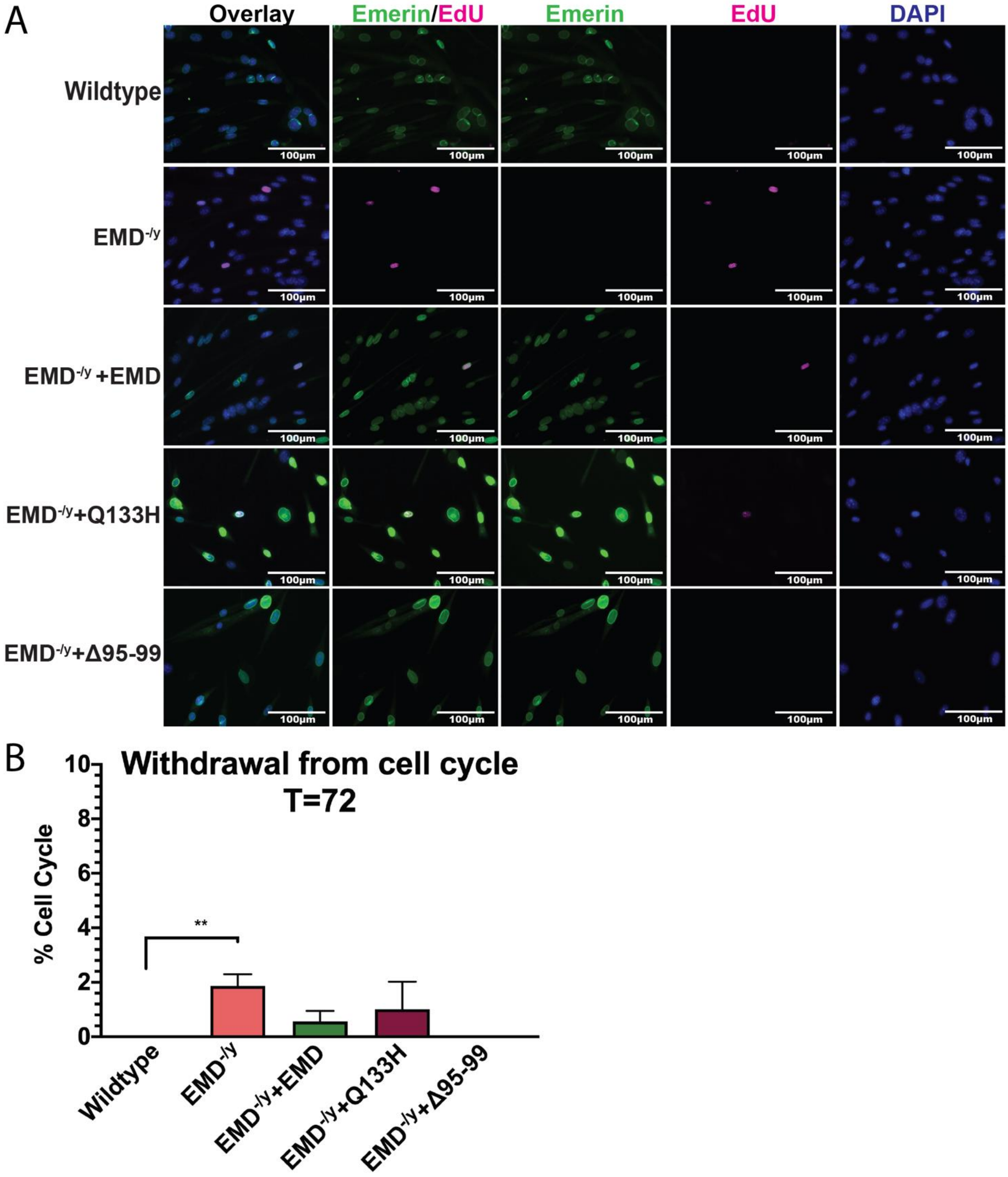

Figure S4. Cell cycle withdrawal 72 h after differentiation induction of EDMD mutant progenitor lines Q133H and Δ95–99. (**A**) Immunofluorescence images at 72 h post differentiation induction. Scale bar: 100 μm. (**B**) The percentage of cells in the cell cycle was calculated for each progenitor line 72 h after differentiation induction. *$p < 0.05$, **$p < 0.02$; #$p < 0.05$ compared to +EMD.

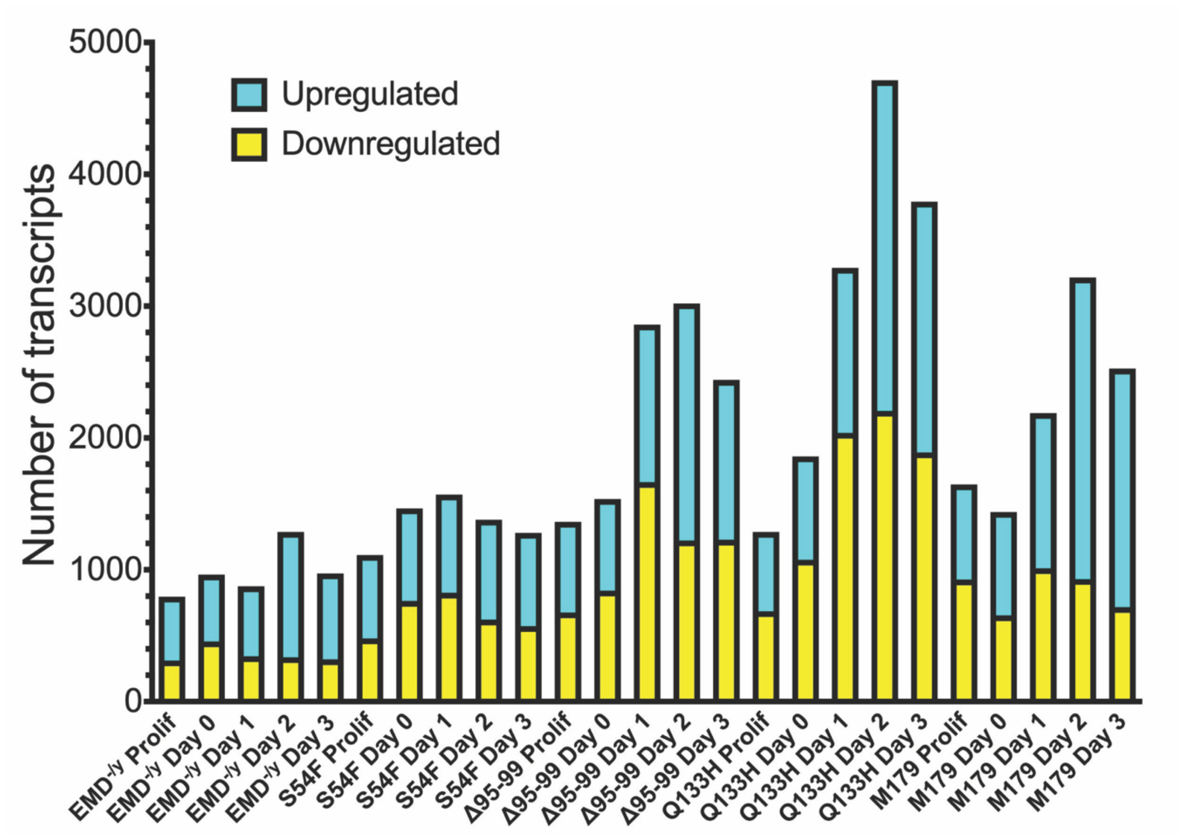

Figure S5. Significant changes in the transcriptome are seen during differentiation of EDMD mutant myogenic progenitors. Differentially expressed transcripts were identified by comparing wildtype progenitors to EMD$^{-/y}$ myogenic progenitors or each EDMD mutant myogenic progenitor during proliferation and each day of differentiation. Blue, upregulated transcripts; Yellow, downregulated transcripts.

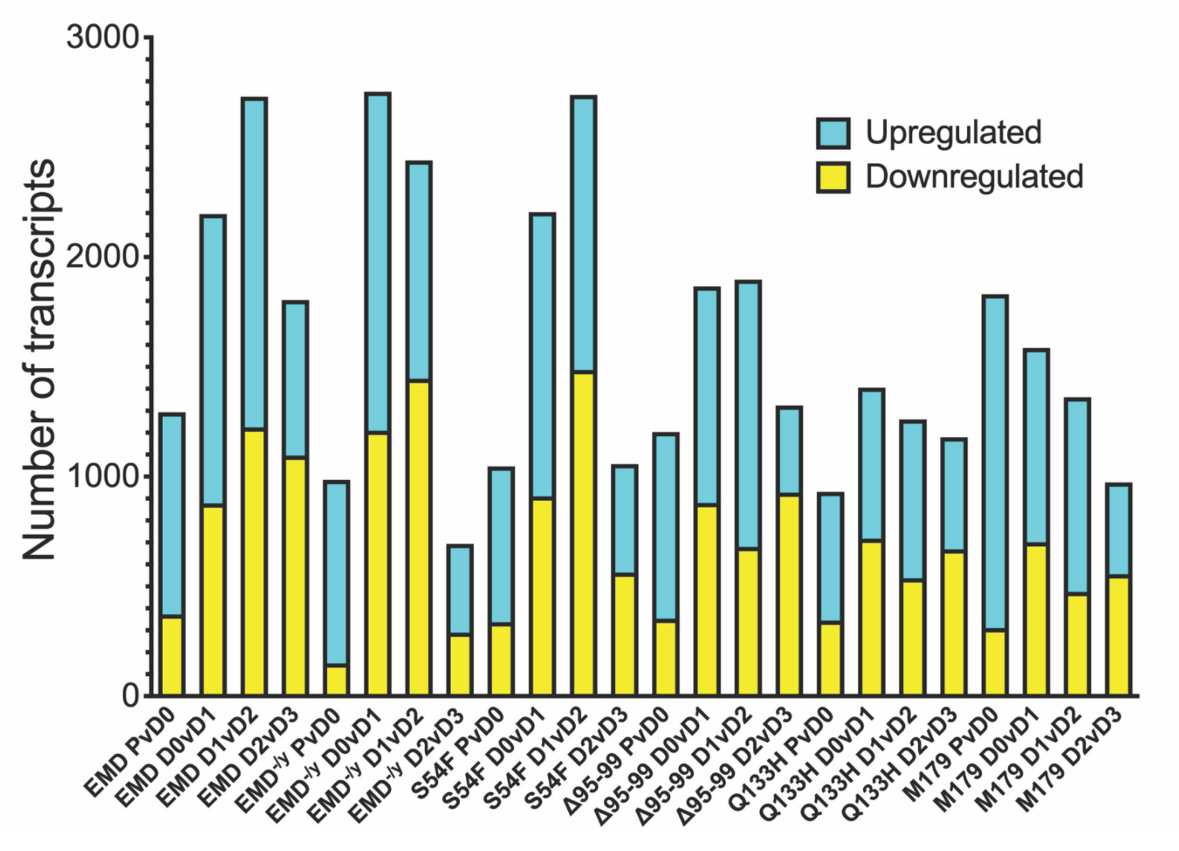

Figure S6. Significant changes in the transcriptome are seen at each daily transition during differentiation of EDMD mutant myogenic progenitors. Differentially expressed transcripts were identified by comparing differentiation day 0 to proliferating cells, differentiation day 1 to differentiation day 0, differentiation day 2 to differentiation day 1, or differentiation day 3 to differentiation day 2 in +EMD cells, EMD$^{-/y}$ cells or each EDMD mutant myogenic progenitor at each transition. Blue, upregulated transcripts; Yellow, downregulated transcripts.
